# PHGDH‐Mediated Serine Metabolism in Nucleus Pulposus Cells Is a Potential Therapeutic Target for Intervertebral Disc Degeneration

**DOI:** 10.1002/advs.78581

**Published:** 2026-10-28

**Authors:** Hao‐Wei Xu, Sheng‐Jie Chang, Xiao‐Wei Liu, Shu‐Bao Zhang, Ying‐Jie Wang, Shan‐Jin Wang

**Affiliations:** ^1^ Department of Spinal Surgery School of Medicine Shanghai East Hospital Tongji University Shanghai China; ^2^ Shanghai Pudong New Area Geriatric Hospital Shanghai China

**Keywords:** autophagy, histone demethylation, intervertebral disc degeneration, phosphoglycerate dehydrogenase, serine

## Abstract

Intervertebral disc degeneration (IVDD) is a major cause of low back pain, driven largely by nucleus pulposus cell (NPC) senescence under chronic hypoxia and nutrient stress. Serine metabolism regulates senescence, but its role in IVDD remains unclear. Here, transcriptomic profiling reveals that the glycine–serine–threonine pathway is downregulated in senescent NPCs, with marked reduction of the serine synthesis rate limiting enzyme PHGDH. This is confirmed by single cell RNA sequencing of human discs, aged rat tissues, and degenerated human samples, accompanied by decreased intracellular serine. PHGDH silencing or serine deprivation inhibits NPC proliferation, induces senescence, and suppresses matrix anabolism. Mechanistically, PHGDH deficiency lowers α ketoglutarate, impairing histone demethylation and increasing H3K27me3 occupancy at the Rap1b promoter, which silences Rap1b and activates mTORC1, thereby blocking autophagic flux. Additionally, serine starvation depletes glutathione, elevates ROS, and promotes apoptosis. In vivo, AAV mediated PHGDH overexpression alleviates puncture induced IVDD, while a serine deficient diet exacerbates degeneration. Collectively, we uncover a PHGDH/Rap1b/mTORC1/autophagy cascade linking serine deprivation to NPC senescence, and propose that restoring PHGDH or serine availability may be a viable strategy to combat age related disc degeneration.

## Graphical abstract

When nucleus pulposus cells senesce, PHGDH decreases, reducing serine synthesis and glutathione, impairing antioxidant capacity. The intermediate α‐KG also drops, lowering JMJD3 demethylase activity, increasing H3K27me3 at the Rap1b promoter, and reducing Rap1b expression, whose down‐regulation in turn activates mTORC1 and suppresses autophagy. Collectively, these events accelerate cellular senescence and ECM degradation, thereby promoting intervertebral disc degeneration.

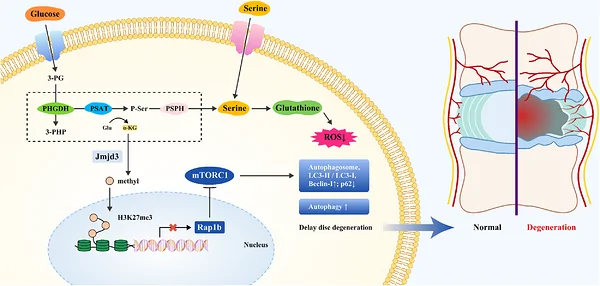

## 1 Introduction

With the intensification of population aging and the transformation of modern lifestyle and working patterns, diseases associated with intervertebral disc degeneration (IVDD) have emerged as the leading cause of low back pain (LBP). Notably, IVDD affects individuals across all age groups, not only severely compromising patients’ quality of life but also imposing a heavy economic burden on families and society at large [1]. Despite extensive research efforts in recent years, the specific pathogenesis of IVDD remains incompletely elucidated. Accumulating evidence has indicated that cellular senescence induced by impaired energy metabolism in nucleus pulposus cells is one of the core mechanisms underlying the initiation and progression of IVDD [2]. Furthermore, multiple factors, including inflammatory responses, oxidative stress, mitochondrial dysfunction, telomere shortening, DNA damage, and epigenetic modifications, have been shown to accelerate the senescence process of nucleus pulposus cells, thereby further exacerbating IVDD progression [3].

To identify differentially expressed genes that are closely associated with nucleus pulposus cell senescence, which may serve as potential targets for IVDD intervention, our research team conducted preliminary experiments. Specifically, nucleus pulposus cells were isolated, and two classic cellular senescence models were established: the replicative senescence (RS) model and the stress‐induced premature senescence (SIPS) model. Subsequently, mRNA transcriptome sequencing and differential gene analysis were performed by comparing these senescent cell models with normal nucleus pulposus cells. Gene set enrichment analysis (GSEA) was further applied to interpret the functional significance of the identified differential genes, and the results demonstrated that the overlapping differentially expressed genes from both RS and SIPS models were significantly enriched in genes involved in glycine, serine, and threonine metabolism during cellular senescence. These key genes include PHGDH, PSAT1, PSPH, and SHMT1/2, among which PHGDH, PSAT1, and PSPH play crucial roles in catalyzing the de novo synthesis of serine derived from glycolytic intermediates.

Serine, as the third most important metabolite involved in cellular metabolism following glucose and glutamate, exerts indispensable effects in a variety of cellular physiological processes. It participates in the biosynthesis of nucleic acids, lipids, amino acids, and prosthetic groups, and is closely correlated with cell proliferation and survival [4]. Accumulating studies have also linked serine metabolism to IVDD progression. For instance, Guo et al. [5] reported that compared with normal intervertebral discs, the differentially abundant metabolites in puncture‐treated degenerative intervertebral discs of rats were significantly enriched in the glycine‐serine‐threonine (Gly‐Ser‐Thr) metabolic pathway, with a marked reduction in the levels of L‐serine, glutathione, and other key metabolites. In another study, Wu et al. [6] comprehensively explored the metabolite profiles at different stages of intervertebral disc degeneration using a combination of nuclear magnetic resonance spectroscopy, histology, cytology, untargeted metabolomics, and mass spectrometry‐based proteomics, and successfully identified key differential metabolic pathways and metabolites. Their findings revealed that a series of metabolic pathways associated with the glycine‐serine‐threonine (Gly‐Ser‐Thr) metabolic axis can regulate carbohydrate metabolism patterns and energy utilization efficiency in nucleus pulposus cells, ultimately delaying IVDD progression through antioxidant effects. Consistent with these previous studies, our previous studies confirmed that serine levels were significantly decreased in senescent nucleus pulposus tissues and cells. We further verified that PHGDH, the rate‐limiting enzyme catalyzing the first step of the serine synthesis pathway (SSP), showed the most prominent differential expression.

Building upon these observations, we hypothesized that PHGDH downregulation and the consequent disruption of serine metabolism are critical drivers of nucleus pulposus cell senescence. In the present study, we therefore sought to systematically investigate the functional role of PHGDH in NPC senescence, delineate the underlying molecular mechanisms, and evaluate the therapeutic potential of targeting the serine synthesis pathway for IVDD.

## 2 Materials and Methods

### 2.1 Patients and Tissue Specimens

The tissue samples were obtained from 8 patients diagnosed with IVDD and 8 patients with burst vertebrae but normal intervertebral discs admitted to our hospital between February 2024 and Dec 2024. All patients underwent lumbar magnetic resonance imaging (MRI) preoperatively, and the Pfirrmann grading system was used to score the degeneration based on disc signal intensity, disc structure, and disc height, categorizing degenerative changes into five grades [7]. Grades I‐II were considered mild degeneration, while grades III‐V were considered severe degeneration. Following discectomy, the intervertebral discs were promptly fixed in 4% paraformaldehyde, followed by embedding and sectioning. Detailed clinical characteristics of the human subjects, including age, sex, BMI, and comorbidities, are provided in Supplementary Table S1. All patients and their families signed informed consent forms, and both human and animal experimentation were approved by the Medical Ethics Committee of Shanghai East Hospital (2022‐2025).

### 2.2 Animals and Experimental Design

24 healthy 2‐month‐old male Sprague‐Dawley (SD) rats were housed in a room maintained at approximately 25°C with a humidity of 60%, under a 12‐h light/12‐h dark cycle. 8 male SD rats were anesthetized via intraperitoneal injection of 3% pentobarbital sodium (30 mg/kg). By palpation, the intervertebral disc levels were identified, with the Co4/Co5 disc designated as the sham group, the Co5/Co6 disc as the puncture+AAV‐NC group, and the Co6/Co7 disc as the puncture + AAV‐PHGDH injection group. To prevent infection, all surgical instruments were sterilely disinfected and the surgical sites were sterilized with iodophor. One week prior to puncture‐induced IVDD, lentivirus was injected in advance. Subsequently, a 21 G puncture needle was inserted into each target intervertebral disc, penetrating fully through the annulus fibrosus, where it was retained and gently rotated 360° for 10 s before being withdrawn [8]. Postoperative radiological assessments were performed at 2, 4 and 8 weeks, while pathological examinations were carried out at the 8‐week time point (Supplementary Table S3). The remaining 16 male SD rats were randomly divided into a control group and a serine‐deficient diet group, with the feed formula designed to be devoid of serine (Supplementary Table S2). After one year of feeding, radiological and pathological assessments were performed to examine lumbar intervertebral disc degeneration (Supplementary Table S4). In addition, to compare the levels of PHGDH and serine in the nucleus pulposus between young and aged rats, eight naturally aged SD rats (24 months old) and eight young SD rats (2 months old) were obtained from Shanghai SLAC Laboratory Animal Co., Ltd.

### 2.3 Imaging Examination

To evaluate the degree of IVDD in rats, both X‐ray and MRI examinations were employed. 3.0 T MRI scanner was used to image the intervertebral discs of all rats, assessing signal and structural changes on T2‐weighted images in the mid‐sagittal plane. The MRI images were imported into ImageJ software, and regions of interest (ROIs) encompassing the intervertebral discs were manually selected on the images. The mean gray value within these ROIs was calculated by averaging the gray values of all pixels. This mean gray value can serve as an indicator of the degree of IVDD in rats, where a lower gray value signifies a higher degree of degeneration [9]. Additionally, X‐ray radiography of the rat vertebrae was conducted to measure changes in the height of the intervertebral space. The height of the intervertebral disc was measured on lateral radiographs by averaging the heights at the anterior, middle, and posterior positions of the disc. Using the same method, the heights of the anterior, middle, and posterior positions of the adjacent vertebrae above and below the disc were also measured and averaged. The ratio of these two averages represents the relative height of the intervertebral disc, also known as the disc height index (as illustrated in Figure 4) [10].

### 2.4 Histological Analysis

The skin, fascia, and muscle layers of the rat's lumbar and tail regions were sequentially dissected to obtain the corresponding segmental vertebral specimens. These specimens were then immersed in a 4% paraformaldehyde solution for 24 h, followed by decalcification in EDTA decalcifying solution for 4 weeks. Subsequently, the specimens were embedded in paraffin and sectioned at a thickness of 5 µm for staining with hematoxylin‐eosin (H&E), safranin O‐fast green (SO&FG), and immunohistochemical techniques. Based on the pathological staining results, histological grading of intervertebral disc degeneration was performed [11], which evaluated aspects such as nucleus pulposus shape, nucleus pulposus area, nucleus pulposus cell count, annulus fibrosus tear, nucleus pulposus‐annulus fibrosus boundary, and endplate ossification. The total score ranged from 0 to 7, with higher scores indicating more severe intervertebral disc degeneration.

### 2.5 Cell Culture and Cellular Senescence Modeling

The isolated rat nucleus pulposus tissues were digested with 0.2% type II collagenase at 37°C for 4–6 h, followed by centrifugation at 1200 rpm for 5 min to remove the supernatant. The tissues were then inoculated into 25 cm^2^ culture flasks using DMEM/F12 medium containing penicillin, streptomycin, and 15% fetal bovine serum. The cells were cultivated in an incubator at 37°C with 5% CO2 by volume, and the medium was changed every 3 days. When the cell confluence rate reached 90%, the cells were digested with 0.25% trypsin and subcultured at a ratio of 1:2. Cells passaged to P10 were considered as senescent rat nucleus pulposus cells. To establish an oxidative stress‐induced senescent nucleus pulposus cells model, P1 nucleus pulposus cells were cultured with 200 µmol/L hydrogen peroxide for 48 h. For PHGDH knockdown, NPCs were transfected with si‐PHGDH1 or si‐NC. Twenty‐four hours after transfection, cells were switched to normal or serine‐free medium and cultured for an additional 48 h (72 h total). We observed that when young nucleus pulposus cells transform into senescent nucleus pulposus cells, their morphology changes from polygonal to elongated spindle forms (fibroblastic changes), and some cells exhibit senescent morphological features such as flattening and increased cell size.

### 2.6 Single‑Cell RNA Sequencing Data Analysis

The scRNA‑seq dataset GSE244889 was downloaded from the GEO database. This dataset contains 7 human intervertebral disc samples with different Pfirrmann grades: 2 samples with grade I, 2 with grade II, 1 with grade III, and 2 with grade IV. We re‑analyzed this dataset to examine PHGDH expression at single‑cell resolution across different cell types in human discs and to track its dynamic changes along the pseudo‑time trajectory of NPC degeneration. Quality control was performed by filtering cells with < 200 or > 5,000 detected genes, > 20% mitochondrial reads, and < 500 unique molecular identifiers (UMIs). The remaining cells were log‑normalized and subjected to dimensionality reduction using principal component analysis (PCA), followed by uniform manifold approximation and projection (UMAP) for visualization. Clustering was performed using the Louvain algorithm. Cell types were annotated with the SingleR package based on reference databases. Nucleus pulposus cells (NPCs) were extracted according to the annotation results.

For pseudo‑time analysis, NPCs were analyzed using Monocle2 to reconstruct developmental trajectories. Differentially expressed genes along pseudo‑time were identified, and the expression dynamics of specific genes (including PHGDH) were visualized.

### 2.7 RNA Sequencing and Bioinformatic Analysis

Total RNA was extracted from rat young (P0), replicative senescent (P5‐P10), H_2_O_2_‐induced senescent NPCs, and control or si‐PHGDH NPCs using TRIzol reagent (Invitrogen). mRNA was enriched, fragmented to ∼300 bp, and subjected to library preparation. Paired‐end (2 × 150 bp) sequencing was performed on an Illumina NovaSeq 6000 platform, generating ∼6 Gb of raw data per sample. Raw reads were filtered by Cutadapt to remove adapters and low‐quality reads (Q20). Clean reads were aligned to the rat reference genome (mRatBN7.2, Ensembl release 107) using HISAT2. Raw read counts per gene were obtained using HTSeq‐count. Differential expression analysis was performed using DESeq2 with raw counts as input, with thresholds of |log_2_FC| > 1 and adjusted p < 0.05. FPKM (Fragments Per Kilobase of transcript per Million mapped reads) values were calculated separately for heatmap visualization only. GO and KEGG enrichment analyses were performed on the overlapping downregulated DEGs from RS and SIPS models using clusterProfiler, and GSEA was performed with default parameters.

### 2.8 Quantitative Real‐Time PCR (qPCR)

Total RNA was reverse transcribed into cDNA using HiScript II first Strand cDNA Synthesis Kit (R211‐01, Vazyme, Nanjing, China). qPCR was performed using SYBR Green Master Mix (11202ES03, Yeasen, Shanghai, China) on a CFX96 Real‐Time System (Bio‐Rad). Gene expression was normalized to GAPDH and calculated using the 2^−^ΔΔCt method. The primer sequences for Phgdh, Psat1, Psph and Gapdh are as shown in the table below.

**Table:** 

	Forward	Reverse
*Rat‐Phgdh*	GCCCACTATGATCGGCCTAC	GTCTCCGTCAGACACCTTGG
*Rat‐Psat1*	AGGCCGTGTTTATGGAGTGG	CCAGTGTCCACAACAGACGA
*Rat‐Psph*	CAGACCCCACTACTACCGGA	ACTGCATCAGCCGAACAGAA
*Rat‐Gapdh*	TTCACCACCATGGAGAAGGC	GGCATGGACTGTGGTCATGA

### 2.9 Western Blotting

Various groups of nucleus pulposus cells were lysed by adding RIPA Lysis Buffer (P0013B, Beyotime, Shanghai, China) containing the protease inhibitor PMSF (ST506, Beyotime, Shanghai, China). After cell lysis, the protein concentration was determined, and 5 × SDS‐PAGE Loading Buffer (P0015, Beyotime, Shanghai, China) was added to the samples. Following treatment, 20 µL of the supernatant was taken for electrophoresis. Electrophoresis was conducted at 80 V in the stacking gel and at 120 V in the separating gel. Proteins were then wet‐transferred to a nitrocellulose membrane at 300 mA for 1.5 h. After membrane blocking, the relevant antibodies for the proteins to be detected were added: PHGDH (14719‐1‐AP, Proteintech, 1:1,000, Wuhan, China), PSAT1 (10501‐1‐AP, Proteintech, 1:1,000, Wuhan, China), PSPH (14513‐1‐AP, Proteintech, 1:1,000, Wuhan, China), Collagen II (86139‐2‐RR, Proteintech, 1:1,000, Wuhan, China), P21 (A19094, Abclonal, 1:1,000, Wuhan, China), Rap1b (A24472, Abclonal, 1:1,000, Wuhan, China), H3K9me3 (39285, Proteintech, 1:1,000, Wuhan, China), H3K9ac (39586, Proteintech, 1:1,000, Wuhan, China), H3K27ac (39685, Proteintech, 1:1,000, Wuhan, China), H3K27me3 (86992‐1‐RR, Proteintech, 1:1,000, Wuhan, China), JMJD3 (55354‐1‐AP, Proteintech, 1:500, Wuhan, China), mTOR (A25581, Abclonal, 1:1,000, Wuhan, China), p‐mTOR (Ser2448; AP0115, Abclonal, 1:500, Wuhan, China), Akt (A18675, Abclonal, 1:1,000, Wuhan, China), p‐Akt (Ser473; AP0637, Abclonal, 1:500, Wuhan, China), P62 (A19700, Abclonal, 1:1,000, Wuhan, China), ATG7 (A19604, Abclonal, 1:1,000, Wuhan, China), Beclin1 (A21191, Abclonal, 1:1,000, Wuhan, China), LC3 (A19665, Abclonal, 1:1,000, Wuhan, China), and GAPDH (A19056, Abclonal, 1:2,000, Wuhan, China), and incubated with oscillation at 4°C overnight. The membrane was washed, and goat anti‐rabbit/mouse IgG secondary antibody (1:2000) was added and incubated at room temperature for 1 h. The protein bands were visualized by chemiluminescence, and images were captured. The ImageJ software was used to analyze the ratio of the grayscale value of the target protein to that of the internal reference protein, and the relative expression level was calculated.

### 2.10 Immunohistochemistry (IHC) and Immunofluorescence (IF)

Paraffin‐embedded rat and human NP tissue sections were deparaffinized, rehydrated, and subjected to antigen retrieval using sodium citrate buffer. For IHC, sections were incubated with primary antibodies against PHGDH and then with biotinylated secondary antibodies, followed by DAB substrate. For IF, cells or tissue sections were permeabilized with 0.1% Triton X‐100, blocked with 5% BSA, and incubated with primary antibodies against H3K27me3, LC3 (green), LAMP1 (red), Collagen II, MMP‐13, and γ‐H2AX. Nuclei were counterstained with DAPI. Prepare the Lyso‐Tracker Red (C1046, Beyotime, Shanghai, China) staining working solution, incubate the cells with it at 37°C for 30 min, and observe lysosomes under a fluorescence microscope. Images were acquired using a confocal microscope (Leica DMI 6000B, Germany). Fluorescence intensity was quantified using ImageJ.

### 2.11 JMJD3 Activity Assay

JMJD3 demethylase activity was measured using the JMJD3 (KDM6B) Chemiluminescent Assay Kit (50406, BPS Bioscience, USA) according to the manufacturer's instructions. Briefly, nuclear proteins were extracted from control and siPHGDH NPCs, and equal amounts of nuclear protein were incubated with the methylated histone H3 peptide substrate precoated on 8‐well strips. After 1 h incubation, primary antibody recognizing the demethylated substrate was added, followed by HRP‐labeled secondary antibody and chemiluminescent substrate. Luminescence was measured using a microplate reader. JMJD3 activity was normalized to total protein input. Total JMJD3 protein levels were examined by Western blot.

### 2.12 Chromatin Immunoprecipitation qPCR (ChIP‐qPCR)

ChIP assays were performed using a BeyoChIP Enzymatic ChIP Assay Kit (P2083S, Beyotime, Shanghai, China) according to the manufacturer's protocol. Briefly, control and si‐PHGDH NPCs were cross‐linked with 1% formaldehyde, and chromatin was sheared by sonication. Immunoprecipitation was performed using an anti‐H3K27me3 antibody or normal rabbit IgG (as a negative control). Purified DNA was analyzed by qPCR using primers spanning the Rap1b promoter region. Enrichment was calculated as a percentage of input. The primers used for ChIP‐qPCR targeting the Rap1b promoter were: forward, 5′‐CGCTCTCCGTTACTTCTCGG‐3′; reverse, 5′‐GCGCGCTGTTCTCCCG‐3′.

### 2.13 Knockdown and Overexpression of PHGDH, Rap1b, and JMJD3

For transfection, rat nucleus pulposus cells (NPCs) were seeded into six‐well plates. When cell density reached approximately 70%, transfection was performed using Lipofectamine 3000 (L3000008, Thermo Fisher Scientific, USA) according to the manufacturer's instructions.

PHGDH and Rap1b were knocked down using siRNAs (Shanghai GenePharma Co., Ltd.). The siPHGDH sequence was 5′‐CAAUGUUGCAGAUGCUCAATT‐3′ (sense) and 5′‐UUGAGCAUCUGCAACAUUGTT‐3′ (antisense). The siRap1b‐1 sequence was 5′‐AAGAAUCUUCUAUCGUAGGAU‐3′ (sense) and 5′‐CCUACGAUAGAAGAUUCUUAU‐3′ (antisense). The siRap1b‐2 sequence was 5′‐UAAGAAUCUUCUAUCGUAGGA‐3′ (sense) and 5′‐CUACGAUAGAAGAUUCUUAUA‐3′ (antisense). Unless otherwise indicated, subsequent experiments after validation were performed using siRap1b‐1.

OE‐PHGDH, OE‐Rap1b, and OE‐JMJD3 plasmids were obtained from Shanghai GenePharma Co., Ltd. (or Genomeditech, Shanghai, China). NPCs were transfected with the indicated overexpression plasmid or empty vector control using Lipofectamine 3000.

AAV‐PHGDH (serotype AAV9) was constructed by Genomeditech (Shanghai, China) using the pAAV‐CMV vector backbone driving rat Phgdh expression, with AAV‐NC as the control, and the viral titer was 1 × 10^10^ pfu/mL. For intradiscal injection, rats were anesthetized, and the tail disc Co5/Co6 or Co6/Co7 was exposed; a total of 2 µL of AAV‐PHGDH or control AAV was injected into the nucleus pulposus using a 33‐gauge needle connected to a 10 µL Hamilton microsyringe, and the injection was performed 7 days before puncture. AAV transduction efficiency and PHGDH overexpression in nucleus pulposus tissues were confirmed by immunofluorescence staining (Supplementary Figure S6A,B).

### 2.14 Cell Proliferation and Viability Assays

Cell proliferation was assessed using the 5‐Ethynyl‐2’‐deoxyuridine (EdU) assay (C0071S, Beyotime, Shanghai, China). NPCs were incubated with 10 µM EdU for 2 h, then fixed and stained according to the manufacturer's protocol. Cell viability was measured using the Cell Counting Kit‐8 (C0037, Beyotime, Shanghai, China). Briefly, cells were cultured in 96‐well plates and 10 µl of CCK‐8 solution was added to each well. After 2 h of incubation, absorbance at 450 nm was measured using a microplate reader.

### 2.15 Senescence‐Associated β‐Galactosidase (SA‐β‐gal) Staining

Cellular senescence was detected using a SA‐β‐gal staining kit (C0602, Beyotime, Shanghai, China). Cells were washed with PBS, fixed with 4% paraformaldehyde for 15 min, and stained with fresh SA‐β‐gal staining solution overnight at 37°C without CO_2_. Stained cells were examined under a light microscope, and the percentage of SA‐β‐gal‐positive cells (blue) was calculated.

### 2.16 Cell Cycle Analysis

After digestion, collect the adherent cells into a centrifuge tube. Add 1 mL of ice‐cold 70% ethanol, gently pipette to mix, and fix at 4°C for 2 h. Prepare an appropriate amount of propidium iodide (PI) staining solution according to the number of samples to be tested (C1052, Beyotime, Shanghai, China). Incubate at 37°C for 30 min in the dark. Detect the cell cycle using a flow cytometer.

### 2.17 Measurement of α‐Ketoglutarate (α‐KG) Levels

Intracellular and tissue α‐KG levels were measured using the Amplex Red α‐Ketoglutarate Assay Kit (S0323S, Beyotime, Shanghai, China) according to the manufacturer's instructions. Briefly, NPC lysates or NP tissue homogenates were deproteinized, and α‐KG was converted to H_2_O_2_ through an enzyme‐coupled reaction. The H_2_O_2_ was then quantified by Amplex Red reagent, and fluorescence was measured at excitation/emission of 571/585 nm. α‐KG concentrations were calculated from a standard curve and normalized to total protein content (for cells) or tissue weight (for tissues).

### 2.18 Α‐KG and GSK‐J4 Treatment

For α‐KG rescue, NPCs were treated with 1 mM dimethyl α‐ketoglutarate (DMKG, Sigma‐Aldrich, USA) for 24 h before harvest. For JMJD3 inhibition, NPCs were treated with 5 µM GSK‐J4 (HY‐15648B, MedChemExpress, USA) for 48 h. Control cells received an equal volume of DMSO.

### 2.19 MHY1485 And Rapamycin Treatment

For mTORC1 activation, NPCs were treated with 5 µM MHY1485 (HY‐B0795, MedChemExpress, USA) for 24 h before harvest. For mTORC1 inhibition, NPCs were treated with 100 nM Rapamycin (HY‐10219, MedChemExpress, USA) for 6 h. Control cells received an equal volume of DMSO.

### 2.20 Measurement of Reactive Oxygen Species (ROS)

Intracellular ROS levels were measured using the fluorescent probe 2’,7’‐dichlorodihydrofluorescein diacetate (Reactive Oxygen Species Assay Kit, S0033S, Beyotime, Shanghai, China). After treatment, NPCs were incubated with 10 µM DCFH‐DA for 30 min at 37°C in the dark. All images were obtained using same microscope (Leica DMI 6000B, Germany). Quantitative analyses were conducted using ImageJ software.

### 2.21 Apoptosis Assay

Cell apoptosis was assessed using the Annexin V‐FITC/PI Apoptosis Detection Kit (C1062S, Beyotime, Shanghai, China). NPCs were harvested, washed twice with PBS, and resuspended in 100 µl binding buffer. Cells were incubated with 5 µl Annexin V‐FITC and 5 µl PI for 15 min at room temperature in the dark. Apoptotic cells were analyzed immediately by flow cytometry. The percentage of early (Annexin V‐FITC+/PI‐) and late (Annexin V‐FITC+/PI+) apoptotic cells was calculated.

### 2.22 Untargeted Metabolomics Analysis

Nucleus pulposus cells were cultured in serine‑deficient or normal medium for 72 h. Cells were harvested, quenched with ice‑cold 80% methanol, and vortexed with extraction buffer (methanol:acetonitrile = 3:1, v/v). After sonication and centrifugation, the supernatant was vacuum‑dried and reconstituted in 50% methanol. Metabolite separation was performed on an ACQUITY UPLC HSS T3 column (2.1 mm × 150 mm, 1.7 µm) using a Thermo Vanquish UHPLC system. The mobile phases consisted of 0.1% formic acid in water (A) and 0.1% formic acid in methanol (B) for positive ion mode, and 0.05% acetic acid in water (C) and 0.05% acetic acid in methanol (D) for negative ion mode. Mass spectrometry was carried out on a Thermo QE HF‐X mass spectrometer with electrospray ionization in both positive and negative ion modes. Raw data were processed by Compound Discovery for peak alignment, retention time correction, and peak area extraction. Metabolite identification was achieved by accurate mass matching (<10 ppm) and MS/MS spectral matching against databases.

After total area normalization, orthogonal partial least‑squares discriminant analysis (OPLS‑DA) was performed. Differential metabolites were defined by variable importance in projection (VIP) > 1 and p‑value < 0.05 (Student's t‑test). Kyoto Encyclopedia of Genes and Genomes (KEGG) pathway enrichment analysis was conducted using hypergeometric test.

### 2.23 Serine Measurement by LC‐MS/MS

Intracellular and tissue serine levels were quantified by LC‐MS/MS. Cultured NPCs were harvested, washed with ice‐cold PBS, and lysed. Frozen rat or human nucleus pulposus tissues were homogenized in ice‐cold PBS. Serine was separated on an ACQUITY UPLC HSS T3 column and detected on a Thermo QE HF‐X mass spectrometer in positive ion mode. Quantification was performed using an external calibration curve of serine standards. Intracellular serine levels were normalized to total protein concentration, and tissue serine levels were normalized to tissue weight.

### 2.24 Bafilomycin A1 Treatment and mRFP‐GFP‐LC3 Assay

NPCs were transfected with the mRFP‐GFP‐LC3 plasmid using Lipofectamine 3000. Twenty‐four hours after transfection, cells were divided into three groups: (i) control, (ii) siRap1b, and (iii) siRap1b + Rapamycin (100 nM, 6 h). Each group was cultured with or without Bafilomycin A1 (Baf A1, 100 nM, 4 h) before fixation. Cells were then fixed with 4% paraformaldehyde, and nuclei were counterstained with DAPI. Images were acquired using a confocal microscope (Leica DMI 6000B). The numbers of GFP^+^RFP^+^ (autophagosomes) and GFP^−^RFP^+^ (autolysosomes) puncta were quantified using ImageJ. The ratio of GFP^−^RFP^+^ to GFP^+^RFP^+^ puncta was used as an indicator of autophagic flux.

### 2.25 Statistical Analysis

The experimental results were analyzed using SPSS 27.0 (IBM, USA) for data analysis and GraphPad Prism 11.0.1 (GraphPad Software, USA) for statistical plotting. Independent sample t‐tests were used for analysis between two groups. While one‐way analysis of variance (ANOVA) was employed for comparison among multiple groups, and that between groups with Tukey's multiple comparisons test. Measurements obtained from different intervertebral discs within the same rat were treated as matched observations. Continuous variables were analyzed using repeated‐measures one‐way ANOVA with Geisser‐Greenhouse correction, followed by Šídák's multiple comparisons test. Histological scores were analyzed using the Friedman test followed by Dunn's multiple comparisons test. Data are presented as individual values with mean ± SD for continuous variables and as individual values with median for histological scores, unless otherwise indicated. All tests were two‐sided, and p < 0.05 was considered statistically significant.

## 3 Results

### 3.1 Disturbed Serine Metabolism and PHGDH Downregulation Characterize Senescent NPCs

At the beginning of this study, we established three NPC models: young (P0), replicative senescent (P5‐P10), and H_2_O_2_‐induced senescent NPCs. We first validated the senescent phenotype of the latter two models using multiple independent assays. SA‐β‐gal staining confirmed a significant increase in senescence‐associated β‐galactosidase activity in both RS and SIPS models (Figure 1A,B). ELISA analysis showed elevated secretion of the SASP factors IL‐1β and IL‐6 in senescent NPCs (Figure 1C,D). Consistently, Western blot analysis revealed increased protein levels of the classic senescence markers P21 and P16 (Figure 1E,F). These data collectively confirm that both senescence models exhibit robust and consistent senescent phenotypes.

**FIGURE 1:**
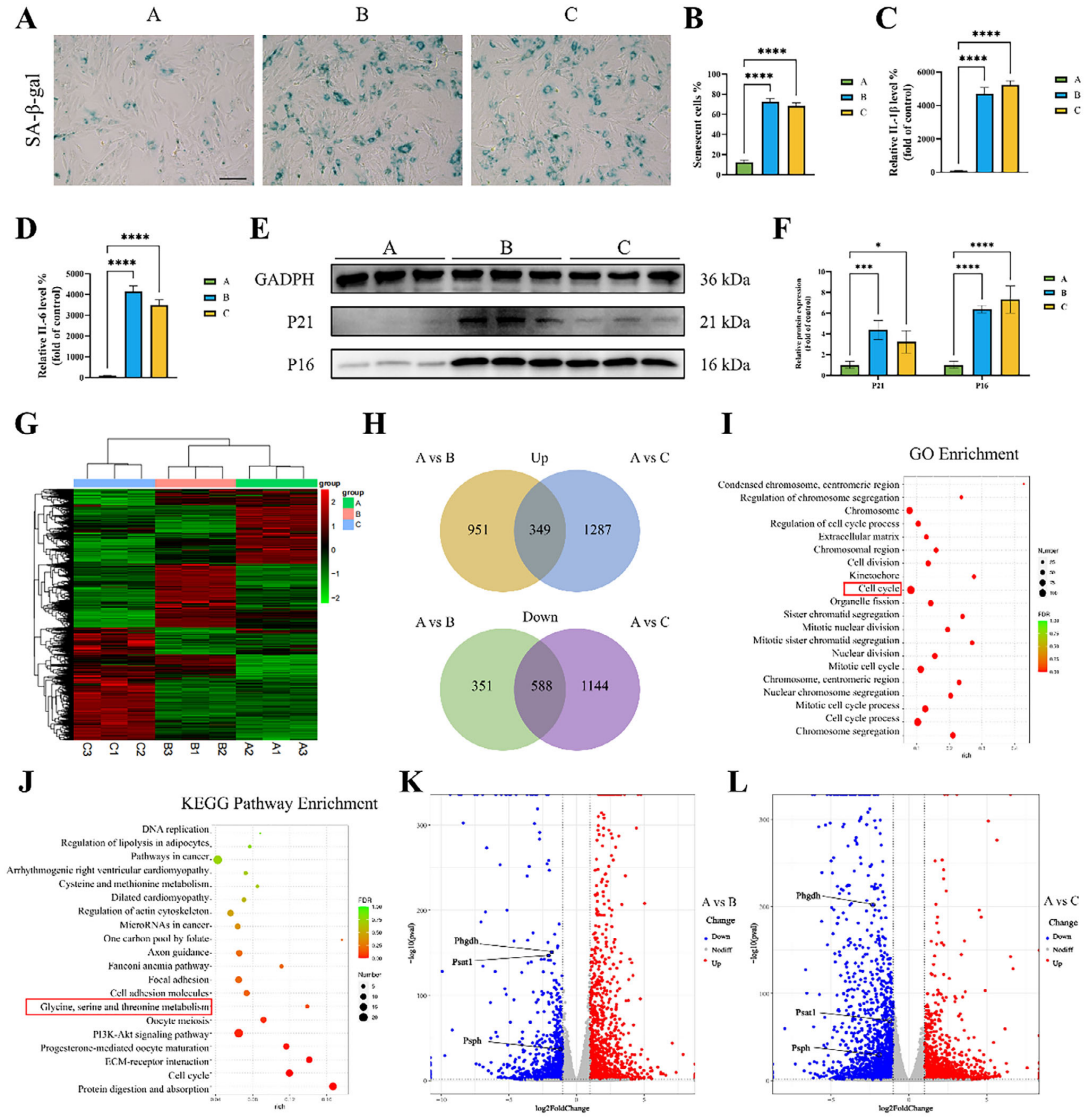
Disturbed serine metabolism occurs in nucleus pulposus cells (NPCs) during senescence. A, B: Representative SA‐β‐gal staining images and quantification of SA‐β‐gal‐positive cells in young NPCs (group A), replicative senescent NPCs (group B), and oxidative stress‐induced senescent NPCs (group C). Scale bar = 50 µm. C, D: ELISA analysis of IL‐1β and IL‐6 secretion in groups A, B, and C (n = 3 per group). E, F: Western blot and quantitative analysis of P21 and P16 expression in groups A, B, and C. G, H: Heatmap and Venn diagram of differentially expressed genes (DEGs) in groups A, B, and C analyzed by RNA‐seq. I, J: The overlapping downregulated genes from groups B and C were intersected, ranked by p‐value and false discovery rate (FDR), and the top 20 enriched pathways were identified. GO analysis revealed significant enrichment in cell cycle‐related pathways (I), whereas KEGG analysis highlighted prominent enrichment in the glycine, serine, and threonine metabolism pathway (J). K, L Volcano plots of DEGs showing that phosphoglycerate dehydrogenase (PHGDH), the rate‐limiting enzyme for serine synthesis, was downregulated in both group B and group C compared with group A. P values were calculated using one‐way analysis of variance (ANOVA) with Tukey's multiple comparisons test. *p < 0.05, **p < 0.01, ***p < 0.001, ****p < 0.0001; ns, not significant.

Transcriptomic profiling of young (P0), replicative senescent (P5‐P10), and H_2_O_2_‐induced senescent NPCs revealed a marked convergence of downregulated genes in the glycine, serine, and threonine metabolism pathway (Figure 1G–J). GO and KEGG enrichment analyses were performed on the overlapping downregulated DEGs from the RS and SIPS models. Volcano plots further identified PHGDH, the rate‐limiting enzyme of the serine synthesis pathway, as prominently suppressed in both senescence models (Figure 1K,L).

To validate these findings in human samples at single‐cell resolution, we re‐analyzed the scRNA‐seq dataset GSE244889 from human intervertebral discs. This dataset contains human intervertebral disc samples from patients with varying Pfirrmann grades, representing a spectrum from healthy to severely degenerated states. Cell type annotation using SingleR identified distinct populations, including nucleus pulposus cells (NPCs) (Figure 2A). Dot plot analysis confirmed that PHGDH was widely expressed across multiple cell types, with notable enrichment in NPCs (Figure 2B–D). Subsequently, pseudo‐time trajectory analysis was performed on NPCs to reconstruct their differentiation/senescence progression. The heatmap of the top 50 genes differentially expressed along pseudo‐time revealed distinct expression clusters, and PHGDH emerged as one of the significantly downregulated genes during the trajectory (Figure 2E). Consistently, the expression dynamics of PHGDH along pseudo‐time showed a progressive decline (Figure 2F), mirroring the reduced PHGDH levels observed in our senescent NPC models. Moreover, the NPC marker gene also exhibited a decreasing trend along pseudo‐time (Figure 2G–I), further corroborating the degenerative phenotype.

**FIGURE 2:**
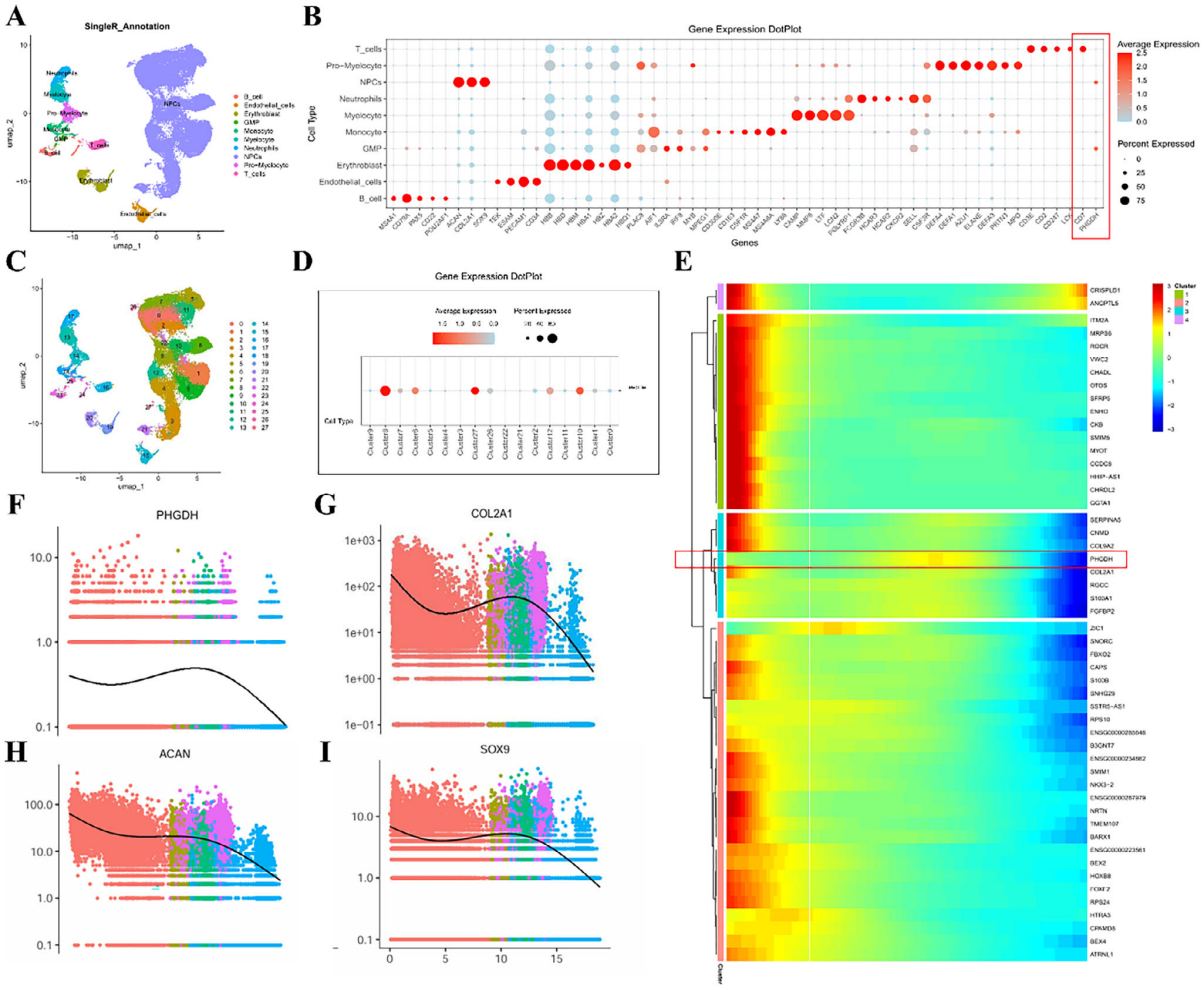
Single‑cell transcriptomic analysis of PHGDH expression in human nucleus pulposus cells. A: UMAP plot showing cell type annotation of human intervertebral disc cells based on the SingleR reference database. Each color represents a distinct cell population. The dataset contains 7 samples with different Pfirrmann grades (2 grade I, 2 grade II, 1 grade III, 2 grade IV). B: Dot plot showing the expression of canonical cell type marker genes and PHGDH across the main cell clusters identified in Figure 2A. Dot size represents the percentage of cells expressing the gene, and color intensity indicates the average expression level. C: UMAP plot of further subdivided cell clusters at higher resolution, revealing 27 distinct subpopulations covering all human intervertebral disc cells. D: Dot plot of PHGDH expression across further subdivided NPC subclusters. E: Heatmap of the top 50 genes differentially expressed along the pseudo‑time trajectory of NPCs. Each column represents a pseudo‑time point, and each row represents a gene. Red: high expression; blue: low expression. F: Pseudo‑time expression dynamics of PHGDH. G‐I: Pseudo‑time expression dynamics of the NPC marker gene.

These transcriptomic findings were further corroborated at the protein and tissue level. qPCR and Western blot analyses showed a progressive loss of PHGDH, PSAT1, and PSPH from P0 to P10, accompanied by reduced Collagen II and elevated P21 expression (Figure 3A–C). Similarly, H_2_O_2_‐induced senescence decreased PHGDH and Collagen II while upregulating P21 (Figure 3D,E). In vivo, PHGDH expression was significantly lower in the nucleus pulposus tissues of aged rats and severely degenerated human discs compared to their young or mildly degenerated counterparts (Figure 3F–J; Supplementary figure S1A,B). Concurrently, intracellular serine levels were substantially reduced in senescent NPCs, H_2_O_2_‐treated cells, aged rat discs, and degenerated human NP tissues (Figure 3K). Having identified PHGDH downregulation as a prominent metabolic feature of senescent NPCs, we next asked whether serine deficiency alone is sufficient to accelerate disc degeneration in vivo.

**FIGURE 3:**
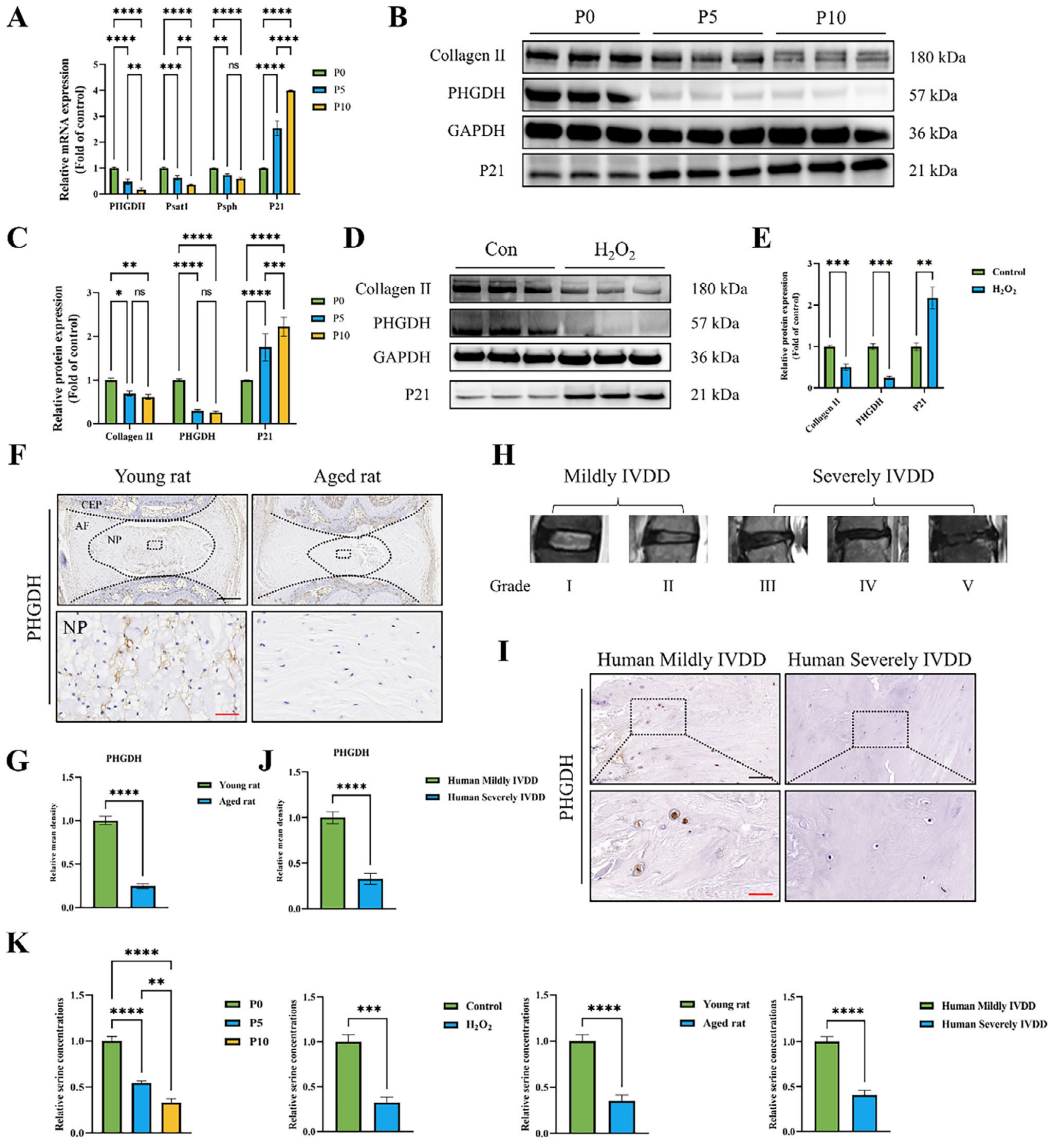
PHGDH expression is markedly decreased in severely degenerated nucleus pulposus cells and tissues. A: Quantitative real‐time PCR (qPCR) analysis of the mRNA expression of serine synthesis‐related enzymes (PHGDH, PSAT1, and PSPH) in primary P0, P5, and P10 NPCs. n = 3 per group. B, C: Western blot and quantitative analysis of Collagen II, PHGDH, and P21 expression in primary P0, P5, and P10 NPCs. n = 3 per group. D, E: Western blot and quantitative analysis of Collagen II, PHGDH, and P21 expression in control and H_2_O_2_‐induced senescent NPCs. n = 3 per group. F, G: Representative immunohistochemistry (IHC) images and quantitative analysis of PHGDH expression in nucleus pulposus tissues from young and aged rats. Black dashed lines indicate the boundaries of the nucleus pulposus (NP), annulus fibrosus (AF), and cartilaginous endplate (CEP). Scale bars: black = 200 µm, red = 50 µm. n = 8 per group. H: Representative T2‐weighted MRI images of human intervertebral discs with different Pfirrmann grades of degeneration (grades I–II, mildly IVDD; grades III–V, severely IVDD). I, J: IHC detection of PHGDH expression in human intervertebral discs with varying Pfirrmann grades of degeneration. Scale bars: black = 200 µm, red = 50 µm. n = 8 per group. K: Serine levels measured in P0, P5, and P10 NPCs. n = 3 per group; control and H_2_O_2_‐treated NPCs. n = 3 per group; nucleus pulposus tissues from young and aged rats. n = 8 per group; and mildly and severely degenerated human nucleus pulposus tissues. n = 8 per group. All data are presented as mean ± standard error of the mean (SEM). P values were calculated using one‐way analysis of variance (ANOVA) with Tukey's multiple comparisons test. *p < 0.05, **p < 0.01, ***p < 0.001, ****p < 0.0001; ns, not significant.

### 3.2 Serine‐Deficient Diet Exacerbates IVDD In Vivo

To determine whether chronic serine insufficiency could exacerbate disc degeneration in vivo, aged rats were placed on a serine‐deficient diet for 12 months (Figure 4A). X‐Ray and T2‐weighted MRI showed diminished signal intensity and reduced disc height in the serine‐deficient group compared with control diet animals (Figure 4B–E). Histological examination confirmed accelerated degeneration, with loss of proteoglycan and a reduced number of notochord cells in the serine‐deficient group (Figure 4F,G). Quantitative analyses of NP area and cellularity confirmed a significant reduction in NP tissue integrity in the serine‐deficient group compared with controls (Figure 4F). Serum and NP tissue serine levels were significantly reduced in the serine‐deficient diet group compared with the control diet group, confirming that the dietary intervention lowered both systemic and local serine availability (Supplementary figure S2A,B). Body weight and general health status showed no significant differences between the two groups over the 12‐month feeding period (Supplementary figure S2C). Correspondingly, immunofluorescence revealed decreased Collagen II and increased MMP13 and γ‐H2AX expression, confirming the detrimental effect of systemic serine deprivation on disc homeostasis (Figure 4H,I). These in vivo findings prompted us to investigate the molecular mechanism by which PHGDH deficiency and serine depletion drive NPC senescence at the cellular level.

**FIGURE 4:**
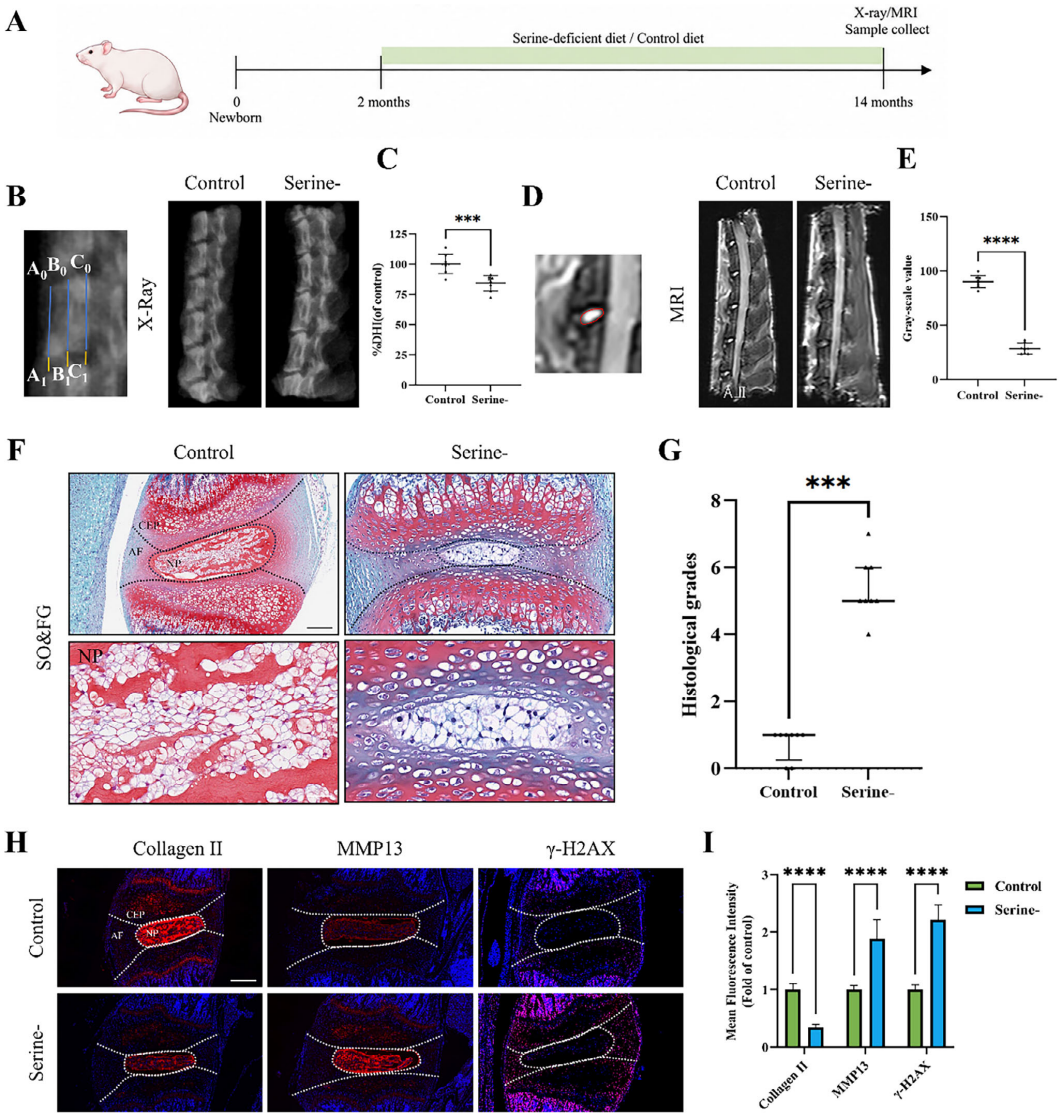
Serine‐deficient diet exacerbates IVDD in aged rats. A: Schematic diagram of the serine‐deficient diet experiment. B, C: Representative X‐ray images and quantitative analysis of intervertebral space height. Disc height index (DHI%) = (A1+B1+C1) × 100%/(A0+B0+C0+A1+B1+C1). n = 8 per group. D, E: Representative T2‐weighted MRI images and quantitative analysis of disc degeneration grades using grayscale values from T2‐weighted MRI. Regions of interest (ROIs) within the intervertebral disc are shown. n = 8 per group. F, G: Representative Safranin O‐Fast Green (SO&FG) staining images and histological scores of disc degeneration. Black dashed lines indicate the NP/AF/CEP boundaries. Scale bars: black = 200 µm, white = 20 µm. n = 8 per group. H, I: Immunofluorescence (IF) staining of IVDD‐related markers in discs from control and serine‐deficient rats and semiquantitative analysis of fluorescence intensity for Collagen II, MMP‐13, and γ‐H2AX. White dashed lines indicate the NP/AF/CEP boundaries. Scale bar = 200 µm. n = 8 per group. P values were calculated using one‐way analysis of variance (ANOVA) with Tukey's multiple comparisons test. *p < 0.05, **p < 0.01, ***p < 0.001, ****p < 0.0001; ns, not significant.

### 3.3 PHGDH Downregulation and Serine Depletion Induce NPC Senescence and Degeneration

A schematic of the serine synthesis pathway is shown in Figure 5A. CCK‐8 assays demonstrated that serine is required for NPC viability in a concentration‐dependent manner (Figure 5B). To determine whether the effect of serine on NPC senescence is concentration‐dependent, we treated NPCs with increasing concentrations of serine (0–200 µM) for 72 h. As shown in Supplementary figure S3, serine deprivation (0 µM) induced the highest levels of SA‐β‐gal positivity and p21 expression, along with the lowest proliferation. As serine concentration increased, SA‐β‐gal positivity and p21 expression progressively decreased, while EdU incorporation and cell viability gradually recovered. At 200 µM, serine restored the phenotype to a level comparable to the normal control. These data demonstrate a concentration‐dependent relationship between serine availability and NPC senescence/proliferation.

**FIGURE 5:**
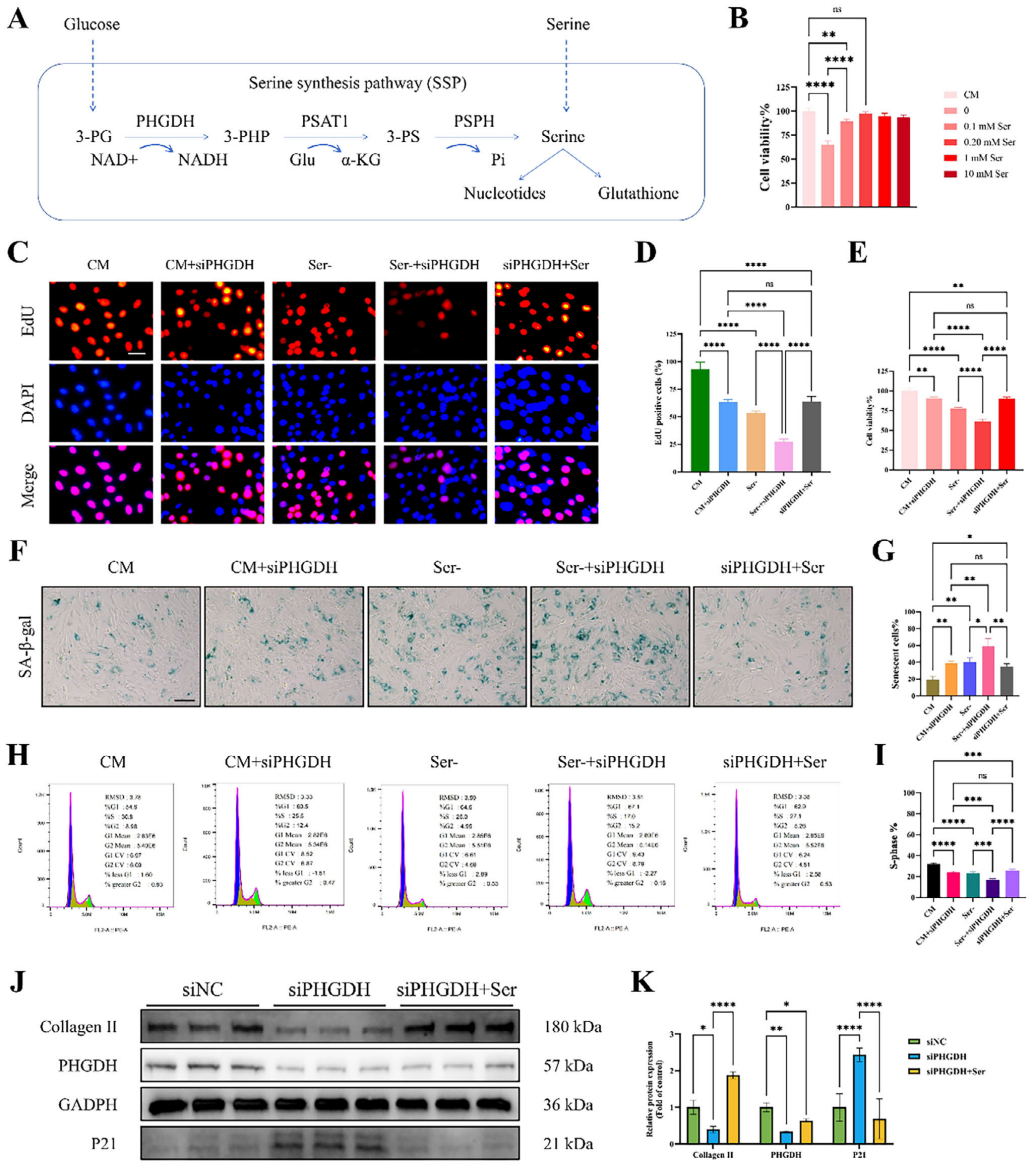
Serine deprivation inhibits proliferation and promotes senescence and degeneration of nucleus pulposus cells. A: Schematic diagram of serine synthesis metabolism. B: Cell Counting Kit‐8 (CCK‐8) assay was performed to evaluate the effects of 0, 0.1, 0.2, 1, and 10 mM serine on NPC viability following 24 h of incubation. n = 3 per group. C, D: NPCs were divided into five groups: complete medium (CM), CM+siPHGDH, serine‐deficient equivalent medium (Ser−), Ser−+siPHGDH, and siPHGDH+Ser, followed by 48 h incubation. 5‐Ethynyl‐2’‐deoxyuridine (EdU) assay was used to detect NPC proliferation. Scale bar = 100 µm. n = 3 per group. E: CCK‐8 assay for viability differences among the five groups. n = 3 per group. F, G: Representative senescence‐associated β‐galactosidase (SA‐β‐gal) staining images and quantitative analysis of SA‐β‐gal activity to assess cellular senescence in the five groups. Scale bar = 50 µm. n = 3 per group. H, I: Propidium iodide staining was applied to analyze cell cycle distribution in each group, with a bar graph showing the statistical proportion of cells in S phase. n = 3 per group. J, K: Western blot and quantitative analysis of Collagen II, PHGDH, and P21 expression in control, siPHGDH, and siPHGDH+Ser NPCs. n = 3 per group. P values were calculated using one‐way analysis of variance (ANOVA) with Tukey's multiple comparisons test. *p < 0.05, **p < 0.01, ***p < 0.001, ****p < 0.0001; ns, not significant.

Both PHGDH knockdown (CM+siPHGDH) and serine deprivation (Ser−) markedly suppressed EdU incorporation and cell viability; the combination showed an additive effect (Figure 5C–E). SA‐β‐gal staining confirmed a strong senescence induction in Ser− and siPHGDH groups, with a further increase upon combined treatment (Figure 5F,G). Cell cycle analysis revealed a significant reduction in the S‐phase population (Figure 5H,I). Exogenous serine supplementation (200 µM) significantly reversed siPHGDH‐induced impairments in cell proliferation and viability, as well as NPC senescence (Figure 5C–I), while also normalizing P21 and Collagen II expression (Figure 5J,K).

### 3.4 PHGDH Deficiency Silences Rap1b Via H3K27me3 Modification

RNA‐seq of control vs. siPHGDH NPCs identified the Rap1 signaling pathway as significantly enriched among downregulated DEGs (Figure 6A,B). Upon PHGDH silencing, both Rap1b protein levels and α‐KG levels in NPCs were significantly decreased, effects that were reversed by exogenous serine supplementation (Figure 6C–E).

**FIGURE 6:**
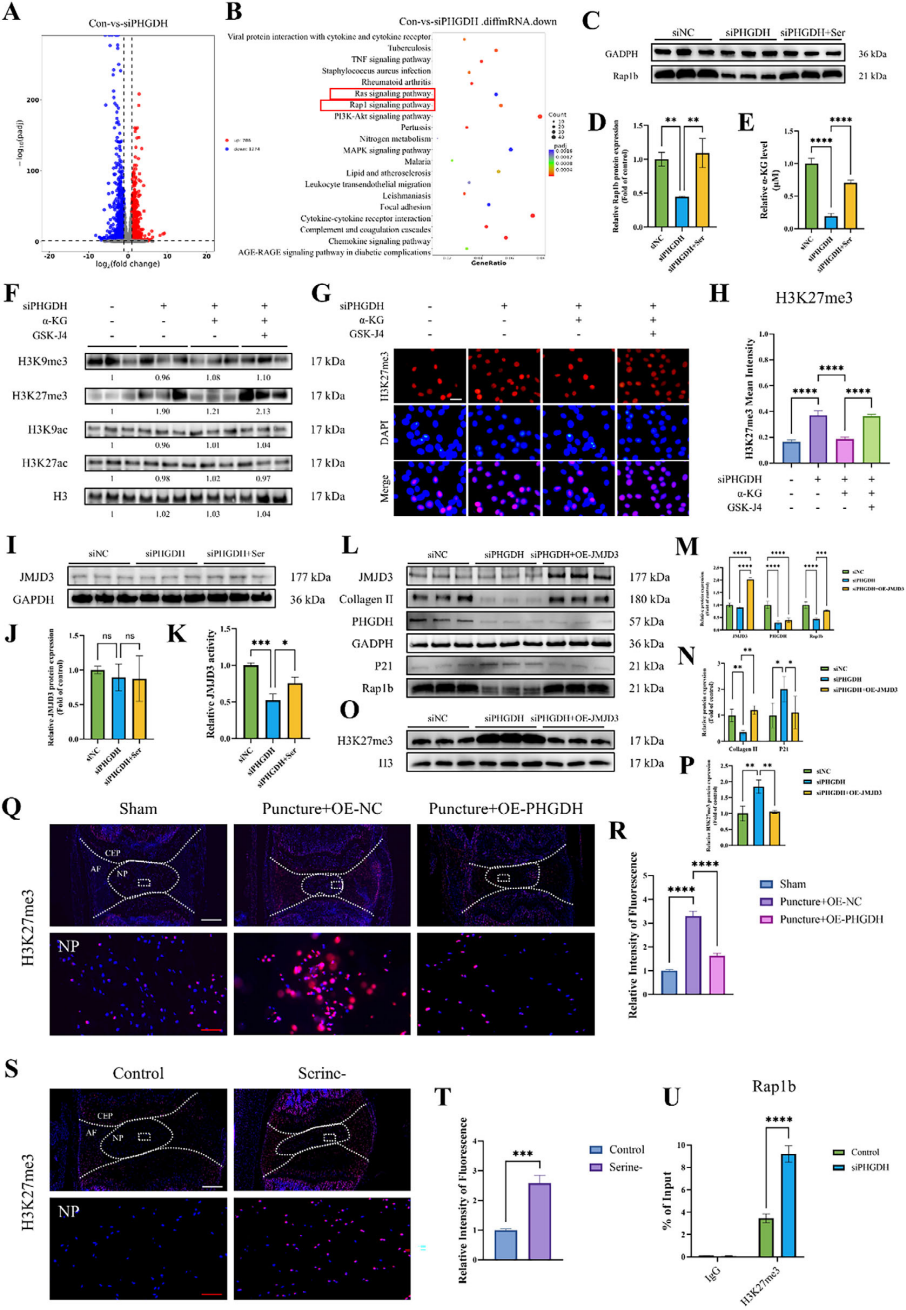
PHGDH modulates Rap1b expression by regulating histone methylation levels at the Rap1b promoter. A, B: Heatmap of DEGs in control and siPHGDH NPCs detected by RNA‐seq and KEGG analysis showing significant enrichment of the Rap1 signaling pathway. C, D: Western blot and quantitative analysis of Rap1b expression in control, siPHGDH, and siPHGDH+Ser NPCs. n = 3 per group. E: Levels of α‐KG in control, siPHGDH, and siPHGDH+Ser groups. n = 3 per group. F: Western blot analysis of H3K9me3, H3K9ac, H3K27ac, and H3K27me3 in control, siPHGDH, siPHGDH+α‐KG, and siPHGDH+α‐KG+GSK‐J4 NPCs (α‐KG: 2 mM; GSK‐J4: 5 µM, 48 h). n = 3 per group. G, H: Differences in H3K27me3 immunofluorescence intensity among the four groups of NPCs. n = 3 per group. I, J: Western blot and quantitative analysis of JMJD3 protein expression in control, siPHGDH, and siPHGDH+Ser NPCs. n = 3 per group. K: Relative JMJD3 activity of NPCs in control, siPHGDH, and siPHGDH+Ser groups. n = 3 per group. L‐N: Western blot and quantitative analysis of JMJD3, PHGDH, Rap1b, Collagen II, and P21 expression in control, siPHGDH, and siPHGDH+OE‐JMJD3 NPCs. n = 3 per group. O, P: Western blot and quantitative analysis of H3K27me3 expression in control, siPHGDH, and siPHGDH+OE‐JMJD3 NPCs. n = 3 per group. Q, R: Differences in H3K27me3 immunofluorescence intensity in rat nucleus pulposus tissues from Sham, puncture+OE‐NC, and puncture+OE‐PHGDH groups. White dashed lines indicate the NP/AF/CEP boundaries (scale bars: white = 200 µm, red = 50 µm). n = 8 per group. S, T: Differences in H3K27me3 immunofluorescence intensity in rat lumbar intervertebral disc tissues from control and serine‐deficient groups. White dashed lines indicate the NP/AF/CEP boundaries (scale bars: white = 200 µm, red = 50 µm). n = 8 per group. U: Chromatin immunoprecipitation qPCR (ChIP‐qPCR) results revealed that PHGDH knockdown increased the occupancy of H3K27me3 at the Rap1b promoter region. n = 3 per group. P values were calculated using one‐way analysis of variance (ANOVA) with Tukey's multiple comparisons test. *p < 0.05, **p < 0.01, ***p < 0.001, ****p < 0.0001; ns, not significant.

To explore the epigenetic mechanism underlying Rap1b silencing, we screened a panel of histone modifications in control and siPHGDH NPCs. PHGDH depletion led to a selective increase in the repressive histone mark H3K27me3, without affecting H3K9me3, H3K9ac, or H3K27ac (Figure 6F). This accumulation was partially reversed by addition of the demethylase cofactor α‐KG (Figure 6F). Consistently, exogenous serine supplementation (200 µM) also reduced the siPHGDH‐induced elevation of H3K27me3 (Supplementary figure S4A,B). Immunofluorescence confirmed elevated H3K27me3 in PHGDH‐deficient NPCs, which was rescued by α‐KG but not by the H3K27me3 demethylase inhibitor GSK‐J4, indicating the pivotal role of α‐KG‐dependent demethylation (Figure 6G,H).

Given that α‐KG serves as an essential cofactor for the histone demethylase JMJD3 (KDM6B), which erases the repressive H3K27me3 mark at target gene promoters, we hypothesized that PHGDH deficiency might impair JMJD3 activity and lead to H3K27me3 enrichment at the Rap1b promoter, thereby silencing Rap1b transcription. Using a chemiluminescent assay, we found that JMJD3 activity was significantly reduced in siPHGDH NPCs compared with control cells, while total JMJD3 protein levels remained unchanged (Figure 6I–K). Importantly, exogenous serine supplementation (200 µM) rescued the reduction in JMJD3 activity induced by PHGDH knockdown. These results provide direct enzymatic evidence that PHGDH‐mediated serine metabolism regulates JMJD3 activity through α‐KG availability.

To directly test whether JMJD3 mediates the epigenetic regulation of Rap1b downstream of PHGDH, we performed a rescue experiment by overexpressing JMJD3 in PHGDH‐knockdown NPCs. As shown in Figure 6L–P, siPHGDH alone increased H3K27me3 levels and decreased Rap1b, P21, and Collagen II expression. Importantly, co‐transfection with OE‐JMJD3 significantly reversed these changes: H3K27me3 elevation was reduced, Rap1b expression was restored, P21 was decreased, and Collagen II was increased. PHGDH remained knocked down in the OE‐JMJD3 group, confirming that the rescue was specifically mediated by JMJD3. These data provide direct genetic evidence that JMJD3 is the key demethylase mediating the PHGDH/α‐KG/H3K27me3/Rap1b axis.

In rat discs, H3K27me3 immunofluorescence was intensified in the puncture group and suppressed by PHGDH overexpression; similarly, a serine‐deficient diet increased H3K27me3 signals in the nucleus pulposus (Figure 6Q–T). ChIP‐qPCR directly demonstrated that PHGDH knockdown significantly enriched H3K27me3 occupancy at the Rap1b promoter (Figure 6U).

### 3.5 Rap1b Loss Activates mTORC1 and Impairs Autophagic Flux

Both siRap1b constructs efficiently silenced Rap1b and recapitulated the senescence phenotype, with decreased Collagen II and elevated P21 (Figure 7A,B). Rap1b knockdown markedly increased phosphorylation of mTOR (Ser2448), indicating that Rap1b functions as an upstream negative regulator of mTORC1; these effects were reversed by the mTOR inhibitor Rapamycin (Figure 7C,D). Western blot analysis of autophagy markers revealed that siRap1b caused accumulation of P62, reduction of ATG7 and Beclin1, and decreased LC3‐II/I ratio, indicating impaired autophagic flux downstream of mTORC1 activation; these changes were reversed upon Rapamycin treatment (Figure 7E,F). Moreover, LysoTracker staining showed a diminished lysosomal compartment in siRap1b cells, which was aggravated by the mTOR agonist MHY1485 and restored by Rapamycin (Figure 7G,H). LC3‐LAMP1 colocalization analysis confirmed a significant impairment of autophagosome‐lysosome fusion in Rap1b‐silenced cells (Figure 7I,J), indicating blocked autophagic flux.

**FIGURE 7:**
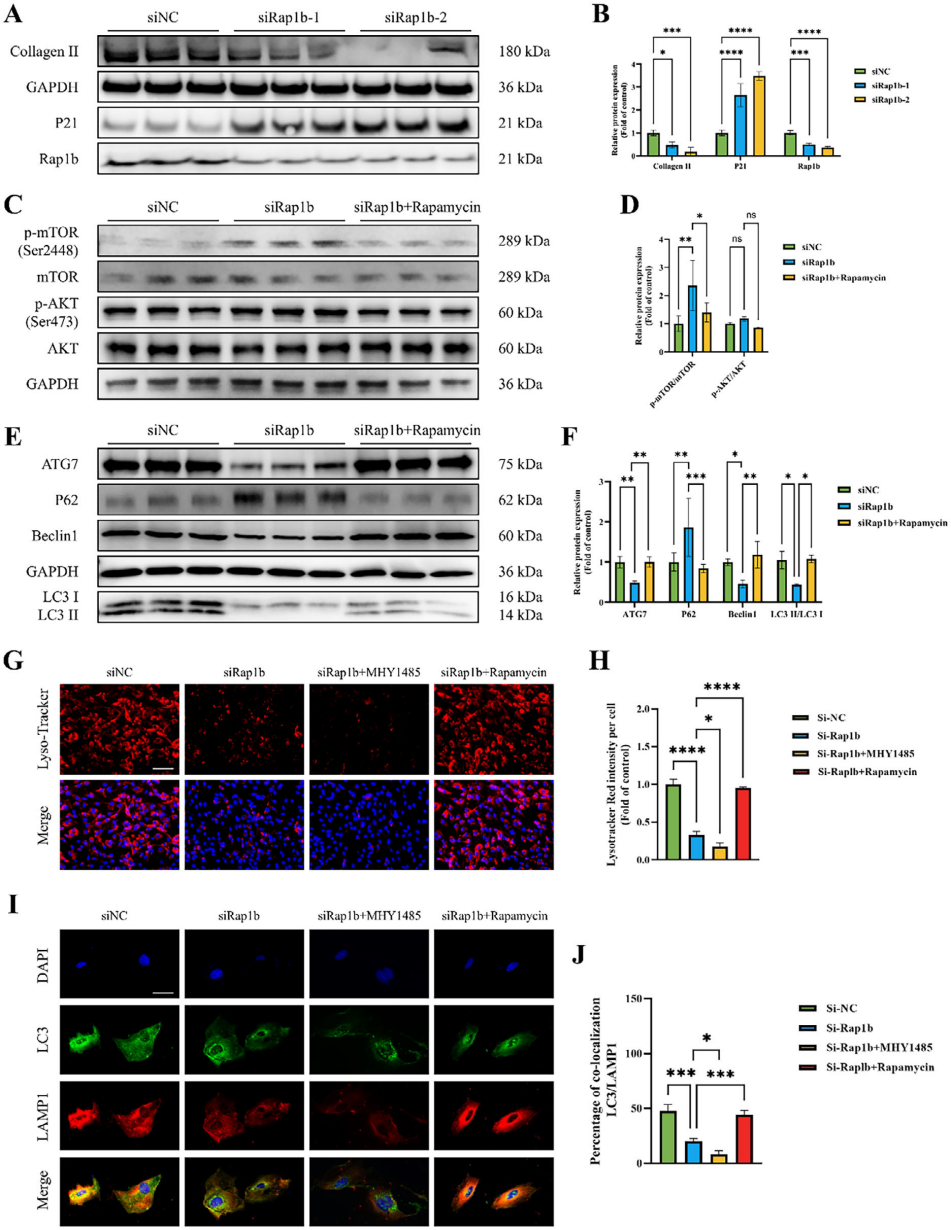
Rap1b silencing activates the mTORC1 pathway and impairs autophagic flux in nucleus pulposus cells. A, B: Western blot and quantitative analysis of Collagen II, P21, and Rap1b expression in control, siRap1b‐1, and siRap1b‐2 NPCs. n = 3 per group. C, D: Western blot and quantitative analysis of phosphorylated mTOR (Ser2448), mTOR, phosphorylated Akt (Ser473), and Akt expression in control, siRap1b, and siRap1b+Rapamycin NPCs. n = 3 per group. E, F: Western blot and quantitative analysis of ATG7, P62, Beclin1, LC3‐I, and LC3‐II expression in control, siRap1b, and siRap1b+Rapamycin NPCs (Rapamycin: 100 nM). n = 3 per group. G, H: LysoTracker staining and quantitative analysis in control, siRap1b, siRap1b+MHY1485, and siRap1b+Rapamycin groups in rat NPCs. Scale bar = 20 µm. n = 3 per group. I, J: Immunofluorescence double‐labeling staining for LC3‐LAMP1 colocalization in rat NPCs (green: LC3; red: LAMP1). Scale bar = 20 µm. The percentage of LC3/LAMP1 colocalization was quantified via image analysis. n = 3 per group. P values were calculated using one‐way analysis of variance (ANOVA) with Tukey's multiple comparisons test. *p < 0.05, **p < 0.01, ***p < 0.001, ****p < 0.0001; ns, not significant.

To further confirm impaired autophagic flux, we performed the mRFP‐GFP‐LC3 tandem fluorescent assay combined with Bafilomycin A1 (Baf A1) lysosomal inhibition. Without Baf‐A1, siRap1b decreased both yellow autophagosome and red autolysosome puncta with an unchanged yellow/red ratio; rapamycin rescued this phenotype. Following Baf‐A1 treatment to block lysosomal degradation, yellow puncta accumulation was attenuated in siRap1b cells, while rapamycin restored the puncta accumulation to levels similar to si‐NC (Supplementary figure S5A‐B). These findings suggest that Rap1b knockdown inhibits autophagy initiation through mTORC1 activation, without impairing late‐stage autophagic degradation.

Consistent with the LysoTracker results, MHY1485 further enhanced mTORC1 activation and aggravated the autophagy impairment in siRap1b cells, as evidenced by increased p‐mTOR, elevated P62, and decreased ATG7, Beclin1, and LC3‐II/I ratio (Supplementary figure S5C–F).

To directly test whether Rap1b mediates the downstream effects of PHGDH, we performed a rescue experiment by overexpressing Rap1b in PHGDH‐knockdown NPCs. As shown in Supplementary figure S5G–J, Rap1b overexpression significantly reversed the effects of siPHGDH: p‐mTOR levels were reduced, P62 accumulation was decreased, the LC3‐II/I ratio was restored, P21 expression was decreased, and Collagen II expression was increased. These results demonstrate that Rap1b functions as a critical downstream mediator of PHGDH in regulating mTORC1 signaling, autophagy, and NPC senescence.

To further establish that Rap1b is a required downstream mediator of PHGDH, we performed a reciprocal rescue experiment by knocking down Rap1b in PHGDH‐overexpressing NPCs. As shown in Supplementary figure S5K,L, OE‐PHGDH alone restored autophagic flux (decreased P62, increased LC3‐II/I) and reduced P21. Importantly, co‐transfection with siRap1b largely abolished these protective effects, with re‐elevation of P62 accumulation, decreased LC3‐II/I ratio, and increased P21. These reciprocal rescue experiments demonstrate that Rap1b is a critical downstream mediator of PHGDH in regulating mTORC1 signaling, autophagy, and NPC senescence.

### 3.6 Serine Deprivation Triggers Redox Imbalance and Apoptosis

Untargeted metabolomics of NPCs cultured in Ser− medium for 72 h showed a prominent decrease in reduced glutathione (GSH), with pathway enrichment again converging on glycine, serine, and threonine metabolism, cell growth and death (Figure 8A–C). Given that GSH is a major ROS scavenger and its synthesis depends on serine‐derived glycine, we next examined whether serine deprivation leads to ROS accumulation. Both PHGDH silencing and serine deprivation significantly increased intracellular ROS levels, and the combination further exacerbated oxidative stress (Figure 8D,E). Flow cytometry with Annexin V/PI staining revealed a marked elevation in apoptosis in siPHGDH and Ser− groups, with an additive effect in the double‐hit condition (Figure 8F,G).

**FIGURE 8:**
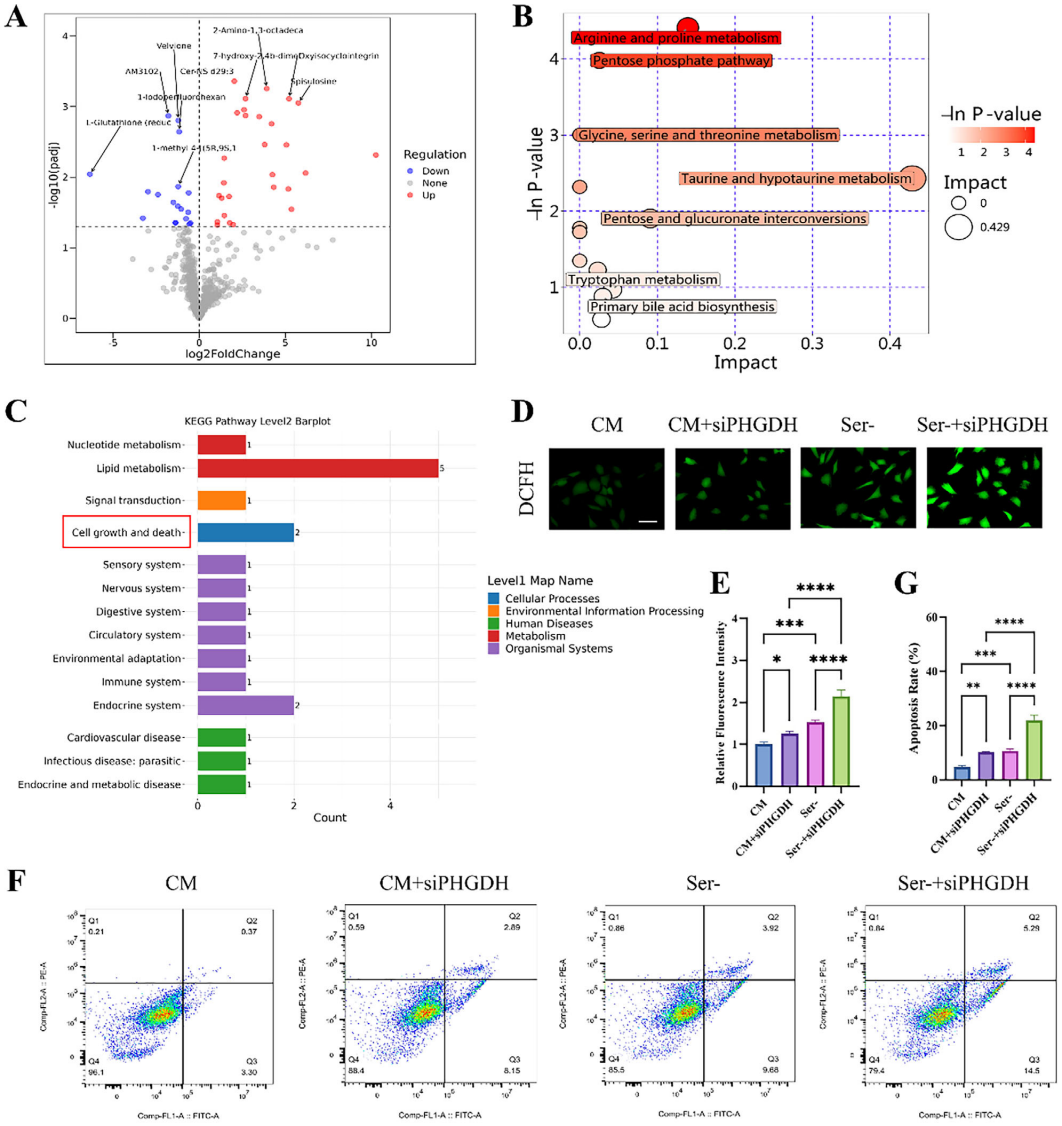
Serine deprivation reduces antioxidant capacity and increases apoptosis in nucleus pulposus cells. A: Volcano plot of differential metabolites in NPCs cultured in serine‐deficient medium (Ser‐) for 48 h, showing decreased L‐reduced glutathione in the Ser‐ group. B: Metabolic pathway enrichment analysis indicated significant enrichment of the glycine‐serine‐threonine metabolism pathway. C: KEGG pathway analysis revealed enrichment in cell growth and death‐related pathways. D, E: Reactive oxygen species (ROS) fluorescence staining and quantitative analysis in control, siPHGDH, Ser‐, and siPHGDH+Ser‐ groups. n = 3 per group. F, G: Apoptosis of NPCs was assessed by Annexin V‐FITC/PI double staining combined with flow cytometry analysis. n = 3 per group. P values were calculated using one‐way analysis of variance (ANOVA) with Tukey's multiple comparisons test. *p < 0.05, **p < 0.01, ***p < 0.001, ****p < 0.0001; ns, not significant.

### 3.7 PHGDH Overexpression Attenuates Puncture‑Induced IVDD In Vivo

In the puncture‐induced IVDD model performed in young adult rats (2‐month‐old), AAV‐mediated PHGDH overexpression was administered locally (Figure 9A). The experimental groups were defined as follows: sham group (anesthesia without puncture), puncture+OE‐NC group, and puncture+OE‐PHGDH group. AAV transduction and PHGDH overexpression in the nucleus pulposus were confirmed by RFP fluorescence and PHGDH immunostaining, respectively (Supplementary figure S6A, B), demonstrating successful local delivery and target gene expression in vivo. To confirm that AAV‐PHGDH overexpression enhanced local serine synthesis, we measured serine levels in NP tissues. As shown in Supplementary figure S6C, local serine levels in NP tissues were significantly elevated in the OE‐PHGDH group compared with the OE‐NC group. X‐ray disc height index (DHI%) was preserved by PHGDH overexpression. T2‐weighted MRI showed that the puncture+OE‐PHGDH group exhibited significantly higher disc grayscale values compared to the puncture‐only group, indicating better hydration (Figure 9B,C). Safranin O‐fast green staining revealed a more maintained extracellular matrix structure and lower histological degeneration scores in the OE‐PHGDH group (Figure 9D,E). Immunofluorescence demonstrated increased Collagen II and decreased MMP‐13 and γ‐H2AX in the PHGDH‐treated discs (Figure 9F,G). In the puncture group, injury markers were broadly distributed across the disc because needle puncture damages both NP and AF. In contrast, in the puncture+OE‐PHGDH group, PHGDH overexpression partially preserved NP tissue and reduced the extent of MMP‐13 and γ‐H2AX positivity, consistent with attenuated disc degeneration.

**FIGURE 9:**
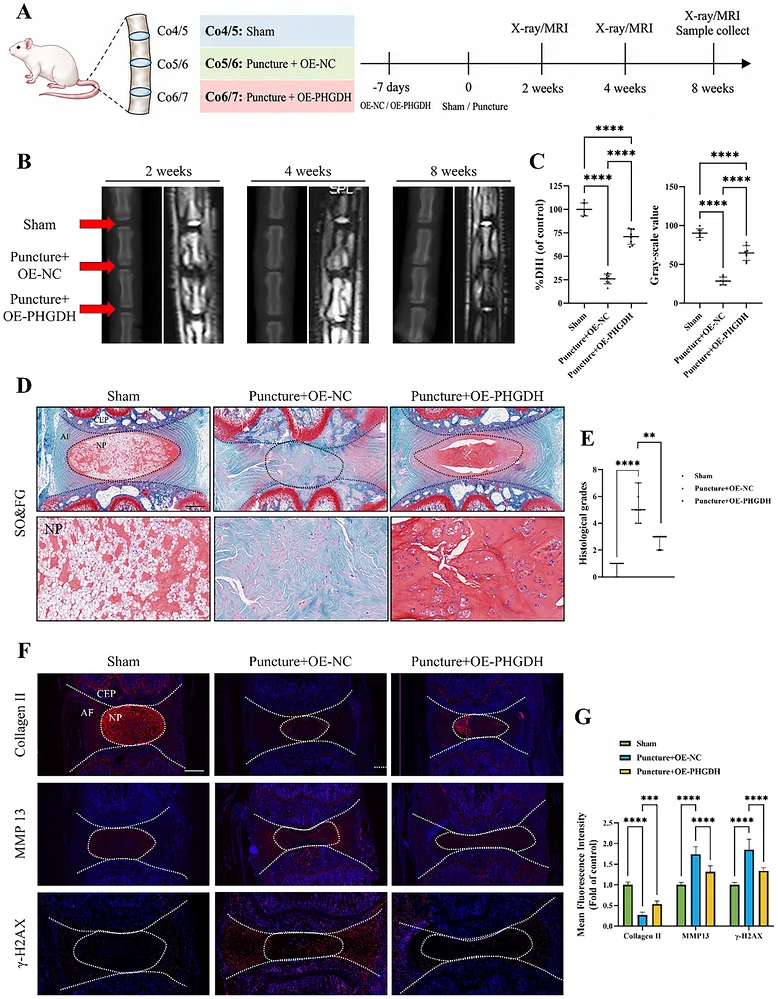
PHGDH overexpression alleviates intervertebral disc degeneration in vivo. A: Schematic diagram of interventions after rat intervertebral disc puncture. B: Representative X‐ray and MRI images. Scale bar = 150 µm. C: Quantitative analysis of intervertebral space height and disc degeneration grades using grayscale values from T2‐weighted MRI images (n = 8 per group). Scale bar = 150 µm. D, E: Representative Safranin O‐Fast Green (SO&FG) staining images and histological scores of disc degeneration. Black dashed lines indicate the NP/AF/CEP boundaries. Scale bars: black = 200 µm, white = 50 µm. n = 8 per group. F, G: IF staining of IVDD‐related markers in discs from Sham, Puncture+OE‐NC, and Puncture+OE‐PHGDH rats and semiquantitative analysis of fluorescence intensity for Collagen II, MMP‐13, and γ‐H2AX. White dashed lines indicate the NP/AF/CEP boundaries. Scale bar = 200 µm. n = 8 per group. P values were calculated using one‐way analysis of variance (ANOVA) with Tukey's multiple comparisons test. Histological degeneration scores are presented as median with interquartile range and were analyzed using the Friedman test, followed by pairwise Wilcoxon matched‐pairs signed‐rank tests with Holm adjustment. *p < 0.05, **p < 0.01, ***p < 0.001, ****p < 0.0001; ns, not significant.

## 4 Discussion

IVDD often leads to low back pain, with senescence of NPCs being one of the significant factors contributing to this process. Unlike other cells, NPCs reside in a hypoxic environment and rely more on glycolysis for energy production [12]. However, the role of glycolysis and amino acid metabolism in the senescence of NPCs remains unclear. In this study, we uncover that PHGDH, the rate‐limiting enzyme of the serine synthesis pathway, acts as a metabolic sentinel that safeguards NPCs against senescence and IVDD. Our findings reveal that the metabolic collapse of the serine pathway is not merely a consequence of senescence but a pivotal driving event that epigenetically silences Rap1b, hyperactivates mTORC1 signaling, and blocks autophagic flux. By delineating a novel PHGDH–Rap1b–mTORC1–autophagy cascade, our work bridges a significant gap between metabolic dysregulation and epigenetic alterations in IVDD pathogenesis.

Serine and its metabolic pathway have emerged as a research hotspot in aging studies in recent years. Serine delays aging at the cellular, organ, and systemic levels through multiple mechanisms, including metabolic reprogramming, epigenetic regulation, antioxidant effects, mitochondrial protection, and inhibition of toxic lipids. Research in this field has advanced from mechanistic validation to animal studies and clinical translational exploration [13]. A randomized, double‐blind, placebo‐controlled trial involving 50 healthy elderly individuals reported that serine supplementation significantly improved spatial memory, learning ability, and problem‐solving performance, with the beneficial effects correlated with elevated plasma serine concentrations [14]. In Caenorhabditis elegans supplemented individually with each of the 20 proteinogenic amino acids, serine and proline exerted the most prominent lifespan‐extending effects, mediated by altered mitochondrial metabolism and activation of the DAF‐16/FOXO and SKN‐1/Nrf2 stress‐response pathways [15]. Furthermore, Yin et al. demonstrated that PHGDH inhibits vascular endothelial cell senescence by regulating the acetylation, stability, and nuclear translocation of pyruvate kinase M2 (PKM2), and endothelial‐targeted overexpression of PHGDH and PKM2 ameliorated vascular senescence in mice [16]. Our results suggest that a serine‐deficient diet accelerates intervertebral disc degeneration in rats, and serine metabolism plays a critical role in nucleus pulposus cell senescence.

PHGDH is one of the three rate‐limiting enzymes in the de novo serine synthesis pathway. Using nicotinamide adenine dinucleotide as a cofactor, PHGDH catalyzes the oxidation of 3‐phosphoglycerate to 3‐phosphohydroxypyruvate, accompanied by the conversion of NAD^+^ to NADH, thereby modulating the cellular redox state [17]. Serine and glycine synthesis is also essential for maintaining cellular redox homeostasis by providing reducing equivalents [18]. Glutathione (GSH), a major redox‐regulating metabolite, is a tripeptide composed of glycine, cysteine, and glutamate that efficiently scavenges reactive oxygen species (ROS) [19]. Mechanistically, serine is a major one‐carbon donor and a precursor for glycine and cysteine, the two rate‐limiting amino acids for GSH synthesis [20]. Serine deprivation therefore limits GSH production, reducing cellular antioxidant capacity and leading to ROS accumulation. In the present study, PHGDH was knocked down in nucleus pulposus (NP) cells, and untargeted metabolomic analysis revealed a remarkable decrease in reduced glutathione levels and a significant increase in intracellular ROS accumulation, consistent with this mechanism. These results suggest that PHGDH may delay IVDD by promoting serine synthesis, increasing GSH production, and regulating intracellular oxidative stress.

Serine availability also influences cellular S‐adenosylmethionine (SAM) levels via de novo ATP synthesis, thereby modulating DNA and RNA methylation [21]. Another methylation‐associated intermediate derived from serine metabolism is α‐ketoglutarate (α‐KG), which serves as a co‐substrate for various histone demethylases [22]. A high α‐KG/succinate ratio promotes the activity of DNA and histone demethylases and is sufficient to remodel multiple chromatin modifications, including histone H3 lysine 27 methylation (H3K27me) [23]. These findings indicate that serine acts as a critical regulator of cellular epigenetics at the DNA, RNA, and protein levels. In our study, targeted metabolomic analysis demonstrated significantly decreased α‐KG levels in PHGDH‐knockdown NPCs, and our previous studies confirmed that α‐KG exerts epigenetic regulatory and anti‐senescence effects, effectively alleviating IVDD and senescence in rat NP cells [10]. Following PHGDH knockdown, RNA‐seq analysis revealed that differentially expressed genes were enriched in the Rap1, Ras, and MAPK signaling pathways, with Rap1b as a key component involved in these cascades.

Furthermore, this study elucidates the specific mechanism by which PHGDH deficiency regulates Rap1b expression through epigenetic modifications. The serine metabolite α‐KG is a critical cofactor for Jumonji C domain‐containing histone demethylases (JMJs) [24], and its level decreases markedly upon PHGDH downregulation or serine deprivation. We found that the reduction in α‐KG directly reduces JMJD3 demethylase activity, as measured by enzymatic assay, while total JMJD3 protein levels remain unchanged. This leads to aberrant enrichment of H3K27me3 at the Rap1b promoter and consequent transcriptional silencing of Rap1b. This finding is consistent with the established mechanism that α‐KG remodels histone methylation marks by modulating JMJ activity, thereby influencing cell fate decisions [25]. Previous studies demonstrated that α‐KG improves the function of senescent bone marrow mesenchymal stem cells by reducing H3K9me3 and H3K27me3 levels and activating BMP signaling and Nanog expression, thereby ameliorating age‐related osteoporosis and promoting bone regeneration in mice [26]. Our findings directly link serine metabolism, histone methylation dynamics, and the transcriptional regulation of a specific gene, providing a new perspective on how the metabolic microenvironment reshapes the chromatin landscape and drives cellular senescence.

Downstream of Rap1b silencing, we delineated the causal relationship between Rap1b, mTORC1, and autophagy. Rap1b functions as an upstream negative regulator of mTORC1. Mechanistically, activated Rap1b sequesters lysosomes in the perinuclear region, limiting the lysosomal surface available for mTORC1 activation and thereby suppressing mTORC1 signaling [27]. Conversely, loss of Rap1b leads to lysosomal dispersion and mTORC1 hyperactivation. Hyperactive mTORC1 then phosphorylates and inhibits ULK1 and other autophagy‐related proteins, blocking autophagosome formation and maturation [28]. Consistently, Rap1b knockdown in our study increased p‐mTOR (Ser2448), reduced the LC3‐II/I ratio, and caused P62 accumulation, indicating impaired autophagic flux. Importantly, pharmacological inhibition of mTORC1 by Rapamycin reversed the autophagy impairment and senescence phenotype induced by Rap1b loss, confirming that Rap1b loss leads to mTORC1 hyperactivation, which in turn impairs autophagic flux and promotes NPC senescence. These results are highly consistent with previous reports that Rap1 regulates lysosomal distribution and mTORC1 activity [29]. Dysregulated mTORC1 signaling is implicated in numerous human diseases, including type 2 diabetes, cancer, obesity, and IVDD [30], and hyperactivation of mTORC1 has been established as a key driver of IVDD, with mTORC1 inhibition enhancing autophagy, alleviating NPC senescence, and reducing matrix degradation [31].

While our study establishes an epigenetic axis linking PHGDH deficiency to mTORC1 hyperactivation through the α‐KG/H3K27me3/Rap1b cascade, we acknowledge that serine deprivation may also regulate mTORC1 through non‐epigenetic mechanisms. First, mTORC1 is directly sensitive to intracellular amino acid availability, particularly leucine and arginine, via the Rag GTPase–Ragulator complex at the lysosomal surface [32]. Serine deprivation could alter the intracellular amino acid pool and thereby affect Rag GTPase‐mediated mTORC1 activation independently of transcriptional or epigenetic changes [33]. Second, serine is a major contributor to one‐carbon metabolism, and its deprivation may affect the SAM/SAH ratio and related metabolic intermediates that influence mTORC1 signaling through methylation‐independent pathways. Third, serine‐derived glycine and cysteine are required for glutathione synthesis, and the resulting redox imbalance may itself modulate mTORC1 activity [34]. Therefore, it is likely that the epigenetic and non‐epigenetic routes act in concert to regulate mTORC1 under serine‐limited conditions, and the relative contribution of each pathway warrants further investigation in future studies.

Taken together with the oxidative stress and GSH depletion induced by serine deprivation in this study, we propose that PHGDH‐mediated serine metabolism cooperatively protects NPC homeostasis through multiple pathways encompassing epigenetics, autophagy, and redox regulation. The novelty of our study lies in the fact that, while previous studies have reported correlative associations between serine metabolism and IVDD, our work is the first to uncover the specific PHGDH–Rap1b–mTORC1–autophagy molecular cascade. This mechanistic framework not only explains how serine metabolic collapse drives NPC senescence but also identifies multiple potential therapeutic nodes for intervention.

## 5 Study Limitations

Several limitations of this study should be acknowledged. First, the human sample cohort was relatively small (n = 8 per group), which may limit statistical power and generalizability; validation in larger, age‐matched cohorts is warranted. Second, although rat models are widely used in IVDD research, anatomical and biomechanical differences between rodent and human discs, particularly notochordal cell persistence and weight‐bearing properties, should be considered. Third, although we confirmed that the serine‐deficient diet significantly reduced both serum and NP tissue serine levels and that body weight and general health status remained comparable between groups, we cannot fully exclude the possibility that subtle systemic metabolic alterations induced by the dietary intervention may contribute indirectly to disc degeneration. Fourth, the in vitro experiments relied on siRNA‐mediated knockdown and exogenous metabolite manipulations, and the senescence models (replicative senescence and H_2_O_2_‐induced senescence) depend strongly on dosage, duration, and culture conditions, which may not fully recapitulate the complex in vivo microenvironment; moreover, the concentrations of serine, α‐KG, and other metabolites used in culture may not precisely reflect physiological levels. Fifth, although we used ACAN, COL2A1, and SOX9 as NPC markers consistent with published single‐cell studies, NPC marker selection remains heterogeneous across the field, and different marker combinations may affect cell identification. Sixth, in this study, PHGDH was only locally overexpressed in the disc for a short period; long‐term systemic PHGDH activation was not induced. Although long‐term serine supplementation was employed, tumorigenesis‐related markers were not examined, and thus the potential tumor‐promoting risk cannot be completely excluded. Future translational studies should prioritize local intradiscal delivery to minimize systemic safety concerns, and cancer screening and risk stratification should be prerequisites before considering systemic serine supplementation. Despite these caveats, our study establishes a conceptual framework linking serine metabolism to NPC senescence and provides a rationale for further translational investigation.

## 6 Conclusion

This study shows that serine was significantly downregulated in senescent NPCs. Knockdown of PHGDH, a key rate‐limiting enzyme in serine synthesis, reduces the serine level of NPCs, weakens the antioxidant stress capacity, reduces the α‐KG level. Further studies show that α‐KG promotes Jmjd3‐dependent H3K27me3 demethylation on the Rap1b promoter to regulate Rap1b expression, thereby regulating NPC autophagy and senescence. Our study identifies the PHGDH–Rap1b–mTORC1–autophagy cascade as a key metabolic node in the pathogenesis of IVDD, and suggests that targeted upregulation of PHGDH or serine supplementation may represent a novel therapeutic strategy to delay intervertebral disc degeneration.

## Author Contributions

**Hao‐Wei Xu**: Conceptualization, Validation, Writing – original draft. **Sheng‐Jie Chang**: Methodology, Project administration, Writing – original draft. **Xiao‐Wei Liu**: Data curation, Formal analysis, Validation. **Shu‐Bao Zhang**: Investigation, Resources. **Ying‐Jie Wang**: Funding acquisition, Supervision. **Shan‐Jin Wang**: Project administration, Supervision, Writing – review & editing. All authors read and approved the final manuscript.

## Ethics Statement

All Animal and Human Experiments Were Reviewed and Approved By the Ethics Committee of Shanghai East Hospital (Approval Number: EC. D (BG).016.02.1, 2022–045).PHGDH‐Mediated Serine Metabolism in Nucleus Pulposus Cells Is a Potential Therapeutic Target for Intervertebral Disc Degeneration.

## Conflicts of Interest

All authors declared no conflicts of interest.

## Supporting information




**Supporting file**: advs78581‐sup‐0001‐SuppMat.docx

## Data Availability

The datasets generated for this study are available on reasonable request to the corresponding author.
