# Supplementary material for: PHGDH‐Mediated Serine Metabolism in Nucleus Pulposus Cells Is a Potential Therapeutic Target for Intervertebral Disc Degeneration
Source: Adv Sci (Weinh). 2026 Oct 28:e78581. Online ahead of print. doi: 10.1002/advs.78581 (PMC13715669; doi:10.1002/advs.78581)
Supplement: Supplementary file 1 [file ADVS-9999-e78581-s001.docx]

**PHGDH-Mediated Serine Metabolism in Nucleus Pulposus Cells Is a Potential Therapeutic Target for Intervertebral Disc Degeneration**

Hao-Wei Xu^1, #^, Sheng-Jie Chang^1, #^, Xiao-Wei Liu^1, #^, Shu-Bao Zhang^1^, Ying-Jie Wang^1, [1]*^, Shan-Jin Wang^1, 2, [2]*^.

**1. Supplementary figures**

**Supplementary figure S1. Western blot validation of PHGDH expression in human nucleus pulposus tissues.**

**A, B:** Representative Western blot and quantitative analysis of PHGDH expression in human nucleus pulposus tissues from patients with mild degeneration (Pfirrmann grades I–II) and severe degeneration (Pfirrmann grades III–V). P values were calculated using one-way analysis of variance (ANOVA) with Tukey’s multiple comparisons test. *P < 0.05, **P < 0.01, ***P < 0.001, ****P < 0.0001; ns, not significant.

**Supplementary figure S2. Serine levels and body weight in rats fed a control or serine-deficient diet.**

**A, B:** Serine levels in serum (A) and nucleus pulposus tissues (B) from rats fed a control or serine-deficient diet for 12 months (n = 8 per group). **C:** Body weight curves of rats in the control and serine-deficient diet groups during the 12-month feeding period (n = 8 per group). P values were calculated using one-way analysis of variance (ANOVA) with Tukey’s multiple comparisons test. *P < 0.05, **P < 0.01, ***P < 0.001, ****P < 0.0001; ns, not significant.

**Supplementary figure S3. Concentration-dependent effects of serine supplementation on nucleus pulposus cell senescence and proliferation.**

**A, B:** Representative SA-β-gal staining images and quantification of SA-β-gal-positive cells in NPCs cultured in serine-free medium supplemented with 0, 50, 100, 150, or 200 μM serine. Scale bar = 50 μm. **C, D:** Representative EdU staining images and quantification of EdU-positive cells in the same groups. Scale bar = 100 μm. **E, F:** Western blot and quantitative analysis of P21 expression in the same groups. P values were calculated using one-way analysis of variance (ANOVA) with Tukey’s multiple comparisons test. *P < 0.05, **P < 0.01, ***P < 0.001, ****P < 0.0001; ns, not significant.

**Supplementary figure S4. Exogenous serine supplementation reverses siPHGDH-induced H3K27me3 elevation.**

**A, B:** Representative Western blot and quantitative analysis of H3K27me3 expression in control, siPHGDH, and siPHGDH+Ser NPCs. P values were calculated using one-way analysis of variance (ANOVA) with Tukey’s multiple comparisons test. *P < 0.05, **P < 0.01, ***P < 0.001, ****P < 0.0001; ns, not significant.

**Supplementary figure S5.** **Autophagic flux analysis and rescue experiments in nucleus pulposus cells.**

**A, B:** Representative confocal images of mRFP-GFP-LC3 tandem fluorescence in NPCs transfected with control, siRap1b, and siRap1b+Rapamycin NPCs, with or without Baf-A1. Relative LC3 puncta represent autolysosomes (red puncta) and autophagosomes (yellow puncta) bar=10 μm. The number of LC3 puncta/cell was calculated. **C, D:** Western blot and quantitative analysis of phosphorylated mTOR (Ser2448), mTOR, phosphorylated Akt (Ser473), and Akt expression in control, siRap1b, and siRap1b+MHY1485 NPCs. **E, F:** Western blot and quantitative analysis of ATG7, P62, Beclin1, LC3-I, and LC3-II expression in control, siRap1b, and siRap1b+MHY1485 NPCs (MHY1485: 5 µM). **G-J:** Western blot and quantitative analysis of phosphorylated mTOR (Ser2448), mTOR, Collagen II, P62, PHGDH, P21, Rap1b, LC3-I, and LC3-II expression in control, siPHGDH, and siPHGDH+OE-Rap1b NPCs. **K, L:** Western blot and quantitative analysis of P62, P21, LC3-I, and LC3-II expression in control, OE-PHGDH, and OE-PHGDH+siRap1b NPCs. P values were calculated using one-way analysis of variance (ANOVA) with Tukey's multiple comparisons test. p<0.05, p<0.01, p<0.001, p<0.0001.

**Supplementary figure S6. Validation of AAV-PHGDH transduction efficiency and local serine levels in rat intervertebral discs.**

**A:** Representative RFP fluorescence images showing AAV9-PHGDH transduction efficiency in the nucleus pulposus of rat intervertebral discs. Scale bar = 200 μm. **B:** Representative immunofluorescence images and semiquantitative analysis of fluorescence intensity for PHGDH expression confirming successful PHGDH overexpression in the nucleus pulposus. Scale bar = 200 μm. **C:** Local serine concentrations in the nucleus pulposus tissues of Sham, Puncture+OE-NC, and Puncture+OE-PHGDH groups (n = 8 per group). P values were calculated using one-way analysis of variance (ANOVA) with Tukey's multiple comparisons test. p<0.05, p<0.01, p<0.001, p<0.0001.

**2. Supplementary table**

**Supplementary Table S1. The basic clinical characteristics of the two groups**

|  | Control (n=8) | IVDD (n=8) | P |
| --- | --- | --- | --- |
| Age, Years | 46.62±9.51 | 52.12±7.84 | 0.209 |
| Sex (M/F) | 5/3 | 4/4 | 0.614 |
| BMI, kg/m^2^ | 24.03±1.44 | 24.47±1.68 | 0.575 |
| Smokers (%) | 3(37.5%) | 2(25.0%) | 0.590 |
| Drinking (%) | 4(50.0%) | 2(25.0%) | 0.302 |
| Hypertension (%) | 2(25.0%) | 3(37.5%) | 0.590 |
| Diabetes (%) | 1(12.5%) | 2(25.0%) | 0.522 |
| Pfirrmann grade |  |  | 0.003 |
| I | 5 (62.5%) | 0 |  |
| II | 3 (37.5%) | 0 |  |
| III | 0 | 2 (25%) |  |
| IV | 0 | 3 (37.5%) |  |
| V | 0 | 3 (37.5%) |  |

**Supplementary Table S2. Composition of the serine-deficient diet**

| Ingredient | kcal/g | gram | kcal |
| --- | --- | --- | --- |
| L-Alanine | 4 | 3.5 | 14 |
| L-Arginine | 4 | 12.1 | 48 |
| L-Asparagine-H_2_O | 4 | 6 | 24 |
| L-Aspartate | 4 | 3.5 | 14 |
| L-Cystine | 4 | 3.5 | 14 |
| L-Glutamic Acid | 4 | 40 | 160 |
| L-Glutamine | 4 | 0 | 0 |
| Glycine | 4 | 0 | 0 |
| L-Histidine-HCl-H_2_O | 4 | 4.5 | 18 |
| L-Isoleucine | 4 | 8 | 32 |
| L-Leucine | 4 | 12 | 48 |
| L-Lysine-HCl | 4 | 18 | 72 |
| L-Methionine | 4 | 8.2 | 33 |
| L-Phenylalanine | 4 | 7.5 | 30 |
| L-Proline | 4 | 3.5 | 14 |
| L-Serine | 4 | 0 | 0 |
| L-Threonine | 4 | 8.2 | 33 |
| L-Tryptophan | 4 | 1.8 | 7 |
| L-Tyrosine | 4 | 5 | 20 |
| L-Valine | 4 | 8 | 32 |
| Dyetrose | 4 | 150 | 600 |
| Cornstarch | 4 | 407.88 | 1631.5 |
| Sucrose | 4 | 100 | 400 |
| Soybean oil | 9 | 80 | 720 |
| TBHQ | 0 | 0.02 | 0 |
| Cellulose | 0 | 50 | 0 |
| Mineral Mix #210050 | 0 | 35 | 0 |
| Calcium phosphate monobasic monohydrate | 0 | 8.2 | 0 |
| Vitamin Mix #310025 | 4 | 13 | 52 |
| Choline Bitartrate | 0 | 2.5 | 0 |
| Blue Dye | 0 | 0.05 | 0 |

**Supplementary Table S3. Experimental design of the AAV-PHGDH therapeutic study in the puncture-induced IVDD model (male SD rats, 2 months old)**

| Group | Day -7 | Day 0 | 2/4 weeks | 8 weeks |
| --- | --- | --- | --- | --- |
| Sham | No treatment | Sham surgery | Imaging | Imaging+sample collection |
| Puncture+OE-NC | Intradiscal AAV-NC injection | Needle puncture |  |  |
| Puncture+OE-PHGDH | Intradiscal AAV-PHGDH injection | Needle puncture |  |  |

**Supplementary Table S4. Experimental design of the serine-deficient diet study (male SD rats, 12 months of feeding)**

| Group | Diet | Duration | Assessment |
| --- | --- | --- | --- |
| Control | Standard control diet | 12 months | Imaging + histological analysis |
| Serine- | Serine-free diet |  |  |
